# Modelling Volumetric Growth in Soft Solids via Residual Stress

**DOI:** 10.1007/s10659-025-10164-2

**Published:** 2025-09-19

**Authors:** Ruoyu Huang, Raymond W. Ogden, Raimondo Penta

**Affiliations:** 1 Lightweight Manufacturing Centre, University of Strathclyde, Renfrew, PA3 2EF UK; 2 School of Mathematics and Statistics, University of Glasgow, Glasgow, G12 8QQ UK

**Keywords:** Residual stress, Volumetric growth, Nonlinear elasticity, 74B20, 74L15

## Abstract

In this paper the nonlinear elasticity theory of volumetric growth based on residual stress that was introduced in previous contribution (Huang et al. in J. Elast. 145:223–241, 2021) is developed further, and is then focused on an applications of the theory with computational examples. The main idea here is to use residual stress in an intact unloaded configuration, or the deformation from a fixed and intact reference configuration (which may itself be residually stressed), as a means to assess the growth in a soft solid, the developing unloaded configuration and the accompanying developing residual stress. The general theory is presented in terms of the free energy per unit mass and the associated energy functions relative to the reference configuration and the unloaded configuration. Growth of a thick-walled spherical shell is examined in order to illustrate the theory using simple prototype energy functions. A general programme for obtaining the developing deformed configuration is outlined and several possible growth laws are discussed for the growth of a spherical shell under internal pressure. This study shows that growth modelling based on the unloaded configurations may provide insights into the development of residual stress and morphology, both of which are, in principle, accessible to experimental observation. For several possible growth laws detailed numerical results are provided to illustrate the evolution of growth and the associated residual stress.

## Introduction

In recent years considerable effort has been expended in developing mechanical theories of growth and remodelling in soft biological tissues. Much of the early work is based on the theory of kinematical growth that was propounded within the framework of nonlinear continuum mechanics in the seminal paper by Rodriguez et al. [1], which generalized the earlier contribution by Skalak and co-workers [2, 3]. Some other representative early theoretical contributions are those by DiCarlo and Quiligotti [4], Lubarda and Hoger [5], Ambrosi and Guana [6], and Garikipati [7].

Applications to the remodelling of artery walls and associated residual stresses include those of Rachev et al. [8], Rachev [9], Taber and Humphrey [10], and Olsson and Klarbring [11]. Extensive reviews of growth, remodelling and morphogenesis were provided by Taber in [12] and [13], the latter concerned, in particular, with the mechanics of the development of the cardiovascular system. Some computational aspects of the modelling of arterial wall growth were developed by Kuhl et al. [14]. Rather than providing extensive references to the literature here, we refer to the reviews on growth and remodelling by Ambrosi et al. [15] and Menzel and Kuhl [16], while the mammoth volume on the mechanics of growth by Goriely [17] contains a wealth of references to many aspects of the subject. Recent advances on general volumetric growth include the works [18–20], and a comparison between different viewpoints on bulk growth mechanics is also provided in [21], while recent works specifically targeted at volumetric avascular tumour growth include for example [22–24].

Residual stresses, which are often present in unloaded configurations of biological tissues, have an important influence on the mechanical behaviour of the tissues, and their role is evident in many of the references cited above. The mathematical framework for the inclusion of residual stress stems from the work of Hoger in several papers, including, for example, [25, 26], which have formed the basis for much subsequent theoretical work, exemplified by Shams et al. [27] and Destrade and Ogden [28]. The interplay between incompatibility-driven growth and residual stresses has also been recently investigated in [29].

In a previous work Huang et al. [30], following a preliminary analysis in Guillou and Ogden [31], discussed a possible mathematical framework for volumetric growth based on the use of residual stress in an unloaded configuration in which the residual stress is growth induced. The motivation for this approach is to avoid using a fictitious stress-free intermediate configuration that is not accessible to experiment.

Growth is a (slow) time-dependent process. This requires development of a novel rate (or incremental) formulation for the unloaded-configuration-based growth theory. Unlike the kinematical growth models based on stress-free intermediate configurations where kinematical variables are used, both the residual stress and kinematical variables are used herein to represent the state of growth in a stressed unloaded configuration. It is then expected that the growth formulation based on the unloaded configurations may provide new insights into growth, especially the interaction between the development of residual stress and that of the evolving morphology. Residual stress is thus included within the constitutive formulation, rather than appearing only as an output from the calculations. The theory developed herein is then exemplified by its application to the growth of an incompressible elastic spherical shell together with numerical illustrations.

The present study is outlined as follows. Section 2 introduces a fixed reference configuration, the evolving unloaded residually stressed configuration and associated kinematics, the residual stress and its governing equations and its boundary conditions. Section 3 develops the constitutive theory for the free energy based on thermodynamic considerations, and specific forms of free-energy function are introduced in Sect. 3.3. The theory is then specialized in Sect. 4 and applied to the problem of a spherical shell subject to internal pressure and either isotropic and anisotropic growth.

Section 5 develops the incremental equations and boundary conditions required for the discussion of ongoing growth, both in general form and for application to the spherical shell, and we introduce a (fairly) general law for describing stress driven growth. In Sect. 6 a possible general programme for updating the residual stress and the evolving unloaded configuration is outlined. This is then followed, in Sect. 7, by an application to the incremental growth of a spherical shell, for which five alternative formulations of the growth law are discussed briefly. In Sect. 8 this theory is applied to the case in which growth is governed by homeostasis.

A method for the numerical implementation of the growth models for an incompressible spherical shell is provided in Sect. 9 and numerical results for several case studies are illustrated in Sect. 10. Some concluding discussion forms the content of Sect. 11.

## Basic Equations

In the following we provide a short summary of the basic equations required for the subsequent analysis. Greater detail can be found in standard texts such as Holzapfel [32] and Ogden [33].

### Description of the Deformation

We consider a continuous body which, at some initial instant, sits in some fixed intact configuration, referred to as the reference configuration, denoted $\mathcal{B}_{0}$. Let material points in $\mathcal{B}_{0}$ be represented by the position vector $\mathbf{X}$. The body, which represents an intact biological tissue, is subject to both deformation and growth and then occupies the deformed configuration ℬ, where the material point $\mathbf{X}$ has become the spatial point $\mathbf{x}$. The connection between $\mathbf{x}$ and $\mathbf{X}$ is described by the mapping $\boldsymbol{\chi} :\mathcal{B}_{0} \to \mathcal{B}$, so that $\mathbf{x} = \boldsymbol{\chi} (\mathbf{X},t) \in \mathcal{B}$, time $t$ being included in the argument here for completeness, although for the most part dependence on $t$ will be implicit. In fact, we emphasize that we shall interpret ‘rate’ as ‘increment’ throughout this paper, where ‘rate’ represents the material time derivative or material increment (at fixed $\mathbf{X}$). Henceforth we omit the explicit dependence on $t$.

The gradient of $\boldsymbol{\chi}$ is the deformation gradient, denoted $\mathbf{F}$ and defined by

1 $$ \mathbf{F}(\mathbf{X}) =\operatorname{Grad}\mathbf{x}= \operatorname{Grad} \boldsymbol{\chi}(\mathbf{X}), $$

$\operatorname{Grad}$ being the gradient operator with respect to $\mathbf{X}$. The determinant $\det \mathbf{F}$, denoted $J$, is strictly positive and represents the ratio of volume elements $\mathrm{d}v/\mathrm{d}v_{0}$, $\mathrm{d}v_{0}$ and $\mathrm{d}v$ being volume elements within $\mathcal{B}_{0}$ and ℬ, respectively.

**Fig. 1:**
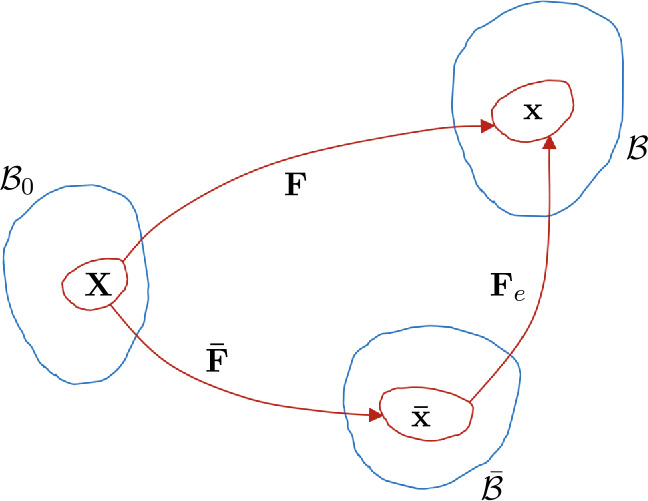
Schematic of the fixed reference configuration $\mathcal{B}_{0}$, the unloaded configuration $\mathcal{\bar{B}}$ and the deformed configuration ℬ (all blue) and the deformation gradients $\mathbf{\bar{F}}$, $\mathbf{F}$ and $\mathbf{F}_{e}$ that connect them. Neighbourhoods of the point $\mathbf{X}$ in $\mathcal{B}_{0}$, $\mathbf{\bar{x}}$ in $\mathcal{\bar{B}}$ and $\mathbf{x}$ in ℬ are shown in red along with red curves connecting them

Suppose, with reference to Fig. 1, that growth is described by the deformation from the configuration $\mathcal{B}_{0}$ into the intact unloaded, but residually stressed, configuration $\mathcal{\bar{B}}$, so that the material point $\mathbf{X}$ moves to the point $\mathbf{\bar{x}}$ with deformation function $\boldsymbol{\bar{\chi}}$ such that $\mathbf{\bar{x}}= \boldsymbol{\bar{\chi}}(\mathbf{X})$ and deformation gradient $\mathbf{\bar{F}}=\operatorname{Grad}\mathbf{\bar{x}}$. On application of mechanical loads $\mathbf{\bar{x}}$ in $\mathcal{\bar{B}}$ becomes $\mathbf{x}$ in ℬ and the associated (elastic) deformation is described by the deformation function $\boldsymbol{\chi}_{e}$ such that $\mathbf{x}=\boldsymbol{\chi}_{e}(\mathbf{\bar{x}})$ with gradient from $\mathcal{\bar{B}}$ to ℬ denoted $\mathbf{F}_{e}$, which is written as $\overline{\operatorname{grad}}\mathbf{x}$, $\overline{\operatorname{grad}}$ being the gradient with respect to $\mathbf{\bar{x}}\in \mathcal{\bar{B}}$. Thus, ℬ is formed from the combination of growth deformation and elastic deformation.

Note, in particular, that it is not our intention to make use of an intermediate stress-free, but disjoint and ill-defined, ‘fictitious’ configuration and an associated growth tensor such as that introduced in Rodriguez et al. [1] and followed by many subsequent authors. Such a configuration is not accessible to experimental observation, and our focus is on the unloaded configuration $\mathcal{\bar{B}}$ as this is in principle accessible to experiment measurement, either directly or indirectly.

When the mechanical loads are removed from the tissue it relaxes back elastically from ℬ to $\mathcal{\bar{B}}$ with inverse deformation gradient $\mathbf{F}_{e}^{-1}$. As growth proceeds both $\mathcal{\bar{B}}$ and the residual stress evolve. While we can imagine that the material in $\mathcal{B}_{0}$ grows into the configuration $\mathcal{\bar{B}}$, this is to allow a convenient theoretical description since growth and elastic deformation are intertwined via the application of loads and in reality growth actually takes place in the loaded configuration ℬ, not in $\mathcal{B}_{0}$.

The local volume ratios associated with the deformation gradients are given by

2 $$ J=\det \mathbf{F},\quad \bar{J}=\det \mathbf{\bar{F}},\quad J_{e}= \det \mathbf{F}_{e}, $$

and the mass densities in the configurations $\mathcal{B}_{0}$, $\mathcal{\bar{B}}$ and ℬ are denoted as $\rho _{0}$, $\bar{\rho}$ and $\rho $, respectively, with the connections

3 $$ \rho _{0}=\rho J=\rho J_{e} \bar{J}=\bar{\rho}\bar{J}. $$

In this paper we consider only materials that are mechanically incompressible so that $J_{e}=1$, $J=\bar{J}$, and $\rho =\bar{\rho}$. Although $\mathcal{B}_{0}$ is a fixed configuration, $\rho _{0}$ is not fixed when growth takes place. We also emphasize that attention is restricted to volumetric growth, so that $\bar{\rho}$ does not change with growth, so that remodelling is not accounted for.

### Governing Equations

In the following we assume, for convenience, that there are no active body forces and, since growth is a slow process and can be treated as quasi-static, inertia terms are omitted. Then, the status of the configuration ℬ is governed by the equilibrium equation

4 $$ \operatorname{div}\boldsymbol{\sigma}=\mathbf{0}\quad \text{in}\ \mathcal{B}, $$

where $\boldsymbol{\sigma}$ is the (symmetric) Cauchy stress tensor generated by loads on the boundary $\partial \mathcal{B}$ and $\operatorname{div}$ is the divergence operator in ℬ. The traction per unit area of $\partial \mathcal{B}$ is denoted $\mathbf{t}$ and is given by

5 $$ \boldsymbol{\sigma}\mathbf{n}=\mathbf{t}, $$

where $\mathbf{n}$ is the unit outward normal to $\partial \mathcal{B}$. Equation (4) may also be written in the pulled-back forms

6 $$ \overline{\operatorname{div}}\mathbf{\bar{S}}=\mathbf{0}\quad \text{in}\ \mathcal{\bar{B}}\quad \text{and}\quad \operatorname{Div}\mathbf{S}=\mathbf{0} \quad \text{in}\ \mathcal{B}_{0}, $$

where $\mathbf{\bar{S}}=\mathbf{F}_{e}^{-1}\boldsymbol{\sigma}$ and $\mathbf{S}=\bar{J}\mathbf{F}^{-1}\boldsymbol{\sigma}$ are the nominal stress tensors on $\mathcal{\bar{B}}$ and $\mathcal{B}_{0}$, respectively, while $\overline{\operatorname{div}}$ and $\operatorname{Div}$ are the divergence operators with respect to $\mathbf{\bar{x}}$ and $\mathbf{X}$, respectively.

In the load-free configuration $\mathcal{\bar{B}}$ the residual stress is denoted $\boldsymbol{\tau}$. This is symmetric and satisfiers the equilibrium equation

7 $$ \overline{\operatorname{div}} \boldsymbol{\tau} = \mathbf{0}\quad \text{in}\ \mathcal{\bar{B}}. $$

As the configuration $\mathcal{\bar{B}}$ is load free $\boldsymbol{\tau}$ satisfies the traction-free condition

8 $$ \boldsymbol{\tau}\mathbf{\bar{n}}=\mathbf{0}\quad \text{on}\ \partial \mathcal{\bar{B}}, $$

$\partial \mathcal{\bar{B}}$ being the boundary of $\mathcal{\bar{B}}$ with unit outward normal $\mathbf{\bar{n}}$. We also define the nominal stress on $\mathcal{B}_{0}$ associated with $\boldsymbol{\tau}$ as $\mathbf{T}=\bar{J}\mathbf{\bar{F}}^{-1}\boldsymbol{\tau}$. This satisfies the equilibrium equation

9 $$ \operatorname{Div}\mathbf{T}=\mathbf{0}\quad \text{in} \ \mathcal{B}_{0}. $$

The different stress tensors defined here will be used in subsequent sections.

The residual stress $\boldsymbol{\tau}$ can be regarded as a measure of growth. Equally, $\mathbf{\bar{F}}$, from which $\boldsymbol{\tau}$ may be calculated by a suitable constitutive equation defined on $\mathcal{B}_{0}$, is a measure of growth.

## Constitutive Equations and Thermodynamic Constraints

Constitutive equations that involve the evolution of growth have to be consistent with the requirements of thermodynamics. If $\mathcal{D}$ denotes the local dissipation rate within the configuration ℬ then, according to standard thermodynamics (see, e.g., [34]), it must be non-negative. This requires

10 $$ \mathcal{D}= \boldsymbol{\sigma}:\mathbf{L} - \rho \hspace{1pt} \dot{\Psi}\geq 0, $$

where $\Psi $ is the free energy in ℬ per unit mass, $\boldsymbol{\sigma}:\mathbf{L} =\operatorname{tr}(\boldsymbol{\sigma} \mathbf{L})$, which identifies the notation: for the double contraction, and here, and subsequently, a superposed dot signifies the material ‘rate’ or increment. The spatial ‘velocity’ gradient $\mathbf{L}=\operatorname{grad}\mathbf{\dot{x}}$ in (10) is given by

11 $$ \mathbf{L} = \mathbf{\dot{F}}\mathbf{F}^{ - 1}. $$

We shall use both free-energy functions $\Psi _{0}$ relative to $\mathcal{B}_{0}$ and $\bar{\Psi}$ relative to $\mathcal{\bar{B}}$.

It should be clarified that we are dealing with growth, which is a dissipative process, within the framework of nonlinear elasticity theory. The dissipation part, related to growth, is reflected in the residual dissipation inequalities summarized in what follows.

### The Free Energy Function $\Psi _{0}$

First, consider the free energy $\Psi _{0}$ per unit volume in $\mathcal{B}_{0}$ given by

12 $$ \Psi _{0}=\rho _{0}\Psi , $$

where $\Psi _{0}$ depends on $\mathbf{F}$ (via $\mathbf{C}=\mathbf{F}^{\mathrm{T}}\mathbf{F}$) and $\rho _{0}$, the latter varying as growth develops from $\mathcal{B}_{0}$ towards $\mathcal{\bar{B}}$. It may also depend on some initial residual stress, say $\boldsymbol{\tau}_{0}$, which is fixed in $\mathcal{B}_{0}$, i.e. it is not an active variable and has the role of a structure tensor. Thus, we write $\Psi _{0}(\mathbf{F},\rho _{0},\boldsymbol{\tau}_{0})$.

On multiplying by $\bar{J}$ the inequality (10) leads to

13 $$ \left (\bar{J}\boldsymbol{\sigma}-\mathbf{F} \frac{\partial \Psi _{0}}{\partial \mathbf{F}}\right ):\mathbf{L}_{e}+ \left (\bar{J}\boldsymbol{\sigma}-\mathbf{F} \frac{\partial \Psi _{0}}{\partial \mathbf{F}}\right ):\mathbf{L}' + \left (\Psi _{0}-\rho _{0} \frac{\partial \Psi _{0}}{\partial \rho _{0}}\right ) \frac{\dot{\rho}_{0}}{\rho _{0}} \geq 0, $$

where $\mathbf{L}$ has been separated into $\mathbf{L}_{e}+\mathbf{L}'$, with $\mathbf{L}_{e}=\mathbf{\dot{F}}_{e}\mathbf{F}_{e}^{-1}$, $\mathbf{L}'=\mathbf{F}_{e}\mathbf{\bar{L}}\mathbf{F}_{e}^{-1}$, and $\mathbf{\bar{L}} =\mathbf{\dot{\bar{F}}}\mathbf{\bar{F}}^{-1} $. Note that $\operatorname{tr}(\mathbf{\bar{L}})=\operatorname{tr}(\mathbf{L}')$. For the considered incompressible material $\mathbf{L}_{e}$ is subject to the constraint $\operatorname{tr}(\mathbf{L}_{e})=0$, with $J_{e}=1$ and $J=\bar{J}$, which requires the introduction of a Lagrange multiplier, here denoted $p$. The Cauchy stress and the corresponding nominal stress (with respect to $\mathcal{B}_{0}$) are then given by

14 $$ \boldsymbol{\sigma}=\bar{J}^{-1}\mathbf{F} \frac{\partial \Psi _{0}}{\partial \mathbf{F}}-p\mathbf{I},\quad \mathbf{S}=\frac{\partial \Psi _{0}}{\partial \mathbf{F}}-\bar{J}p \mathbf{F}^{-1}, $$

while the residual inequality is

15 $$ \left (\Psi _{0}-\rho _{0} \frac{\partial \Psi _{0}}{\partial \rho _{0}}\right ) \frac{\dot{\rho}_{0}}{\rho _{0}}-\bar{J}p\,\operatorname{tr}\mathbf{\bar{L}} \geq 0. $$

When evaluated in $\mathcal{\bar{B}}$, equation (14) gives

16 $$ \boldsymbol{\tau}=\bar{J}^{-1}\mathbf{\bar{F}} \frac{\partial \Psi _{0}}{\partial \mathbf{F}}(\mathbf{\bar{F}}, \rho _{0},\boldsymbol{\tau}_{0})-\bar{p}\mathbf{\bar{I}},\quad \mathbf{T}=\frac{\partial \Psi _{0}}{\partial \mathbf{F}}( \mathbf{\bar{F}}, \rho _{0},\boldsymbol{\tau}_{0})-\bar{J}\bar{p} \mathbf{\bar{F}}^{-1}, $$

thus providing a relation between $\boldsymbol{\tau}$ (or $\mathbf{T}$) and $\mathbf{\bar{F}}$, $\bar{p}$ being the value of $p$ and $\mathbf{\bar{I}}$ the identity tensor in $\mathcal{\bar{B}}$.

Note that when evaluated in $\mathcal{B}_{0}$, equation (16) gives

17 $$ \boldsymbol{\tau}_{0}=\frac{\partial \Psi _{0}}{\partial \mathbf{F}}( \mathbf{I}_{0}, \rho _{0},\boldsymbol{\tau}_{0})-p_{0}\mathbf{I}_{0}, $$

where $\mathbf{I}_{0}$ is the identity tensor in $\mathcal{B}_{0}$ and $p_{0}$ is the corresponding Lagrange multiplier. This compatibility equation provides a connection between $\boldsymbol{\tau}_{0}$, the reference value of $\rho _{0}$ prior to growth, and the form of $\Psi _{0}$. If $\boldsymbol{\tau}_{0}=\mathbf{0}$ then it gives $p_{0}$ in terms of $\rho _{0}$, depending on the form of $\Psi _{0}$.

It is particularly interesting to note the connections

18 $$ \frac{\dot{\rho}_{0}}{\rho _{0}}-\frac{\dot{\bar{\rho}}}{\bar{\rho}}= \frac{\dot{\bar{J}}}{\bar{J}}=\operatorname{tr}(\mathbf{\bar{L}}), $$

which follow from the increments of $\rho _{0}=\bar{\rho}\bar{J}$ and of $\bar{J}=\det \mathbf{\bar{F}}$. As we are assuming that the deformation from $\mathcal{B}_{0}$ to $\mathcal{\bar{B}}$ is purely due to volumetric growth then $\dot{\bar{\rho}}=0$ and the above equation reduces to

19 $$ \frac{\dot{\rho}_{0}}{\rho _{0}}=\frac{\dot{\bar{J}}}{\bar{J}}= \operatorname{tr}(\mathbf{\bar{L}}). $$

Henceforth we have $\dot{\bar{\rho}}=0$ so that dependence on $\rho _{0}$ is equivalent to dependence on $\bar{J}$, and then (15) becomes

20 $$ \left (\Psi _{0}-\rho _{0} \frac{\partial \Psi _{0}}{\partial \rho _{0}}-\bar{J}p\,\right ) \operatorname{tr}\mathbf{\bar{L}}\geq 0. $$

### The Free Energy Function $\bar{\Psi}$

Next, consider $\bar{\Psi}$, per unit volume in $\mathcal{\bar{B}}$, which is given by

21 $$ \bar{\Psi}=\bar{\rho}\Psi =\bar{J}^{-1}\Psi _{0}, $$

and depends on the deformation from $\mathcal{\bar{B}}$ and the residual stress within it. Thus, we have $\bar{\Psi}(\mathbf{F}_{e},\boldsymbol{\tau})$, with the dependence on $\mathbf{F}_{e}$ via the right Cauchy–Green tensor $\mathbf{C}_{e}=\mathbf{F}_{e}^{\mathrm{T}}\mathbf{F}_{e}$.

Similarly to $\Psi _{0}$, by expanding out $\dot{\bar{\Psi}}$, the inequality (10) can be rearranged as

22 $$ \left (\boldsymbol{\sigma}-\mathbf{F}_{e} \frac{\partial \bar{\Psi}}{\partial \mathbf{F}_{e}}\right ): \mathbf{L}_{e} +\boldsymbol{\sigma}:\mathbf{L}' - \frac{\partial \bar{\Psi}}{\partial \boldsymbol{\tau}}: \boldsymbol{\dot{\tau}} \geq 0. $$

In this case the Cauchy stress $\boldsymbol{\sigma}$ and the nominal stress (with respect to $\mathcal{\bar{B}}$) are given by

23 $$ \boldsymbol{\sigma}=\mathbf{F}_{e} \frac{\partial \bar{\Psi}}{\partial \mathbf{F}_{e}}-p\mathbf{I}, \quad \mathbf{\bar{S}}= \frac{\partial \bar{\Psi}}{\partial \mathbf{F}_{e}}-p\mathbf{F}_{e}^{-1}, $$

and the residual inequality is

24 $$ \boldsymbol{\sigma}:\mathbf{L}' - \frac{\partial \bar{\Psi}}{\partial \boldsymbol{\tau}}: \boldsymbol{\dot{\tau}} \geq 0. $$

The inequality (24) imposes a constraint on $\mathbf{L}'$ and $\boldsymbol{\dot{\tau}}$. Its importance is: (1) it is imposed on measurable variables, and (2) it may lead to explicit formulations of growth laws in $\mathcal{\bar{B}}$. Hence (24) reflects the crucial difference of the present modelling from the kinematical models on the fictitious intermediate configuration where the thermodynamic constraint was imposed on the fictitious growth variable and cannot lead either directly or indirectly to contributing to the formulation of growth laws.

Note that when evaluated in $\mathcal{\bar{B}}$, i.e. for $\mathbf{F}_{e}=\mathbf{\bar{I}}$, equation (23) yields

25 $$ \boldsymbol{\tau}=\frac{\partial \bar{\Psi}}{\partial \mathbf{F}_{e}} ( \mathbf{\bar{I}},\boldsymbol{\tau})-\bar{p}\mathbf{\bar{I}}. $$

This provides a connection between $\bar{\Psi}$ and $\boldsymbol{\tau}$ that has to be satisfied in $\mathcal{\bar{B}}$.

The configuration ℬ can be reached directly from $\mathcal{B}_{0}$ or indirectly from $\mathcal{B}_{0}$ to $\mathcal{\bar{B}}$ and then from $\mathcal{\bar{B}}$ to ℬ. The associated energies are related by

26 $$ \Psi _{0}(\mathbf{F},\rho _{0},\boldsymbol{\tau}_{0}) =\Psi _{0}( \mathbf{\bar{F}},\rho _{0},\boldsymbol{\tau}_{0})+\bar{J}\bar{\Psi}^{ \prime}(\mathbf{F}_{e},\boldsymbol{\tau}), $$

with $\mathbf{F}=\mathbf{F}_{e}\mathbf{\bar{F}}$ and $\boldsymbol{\tau}$ given by (16), where, noting (21), $\bar{\Psi}^{\prime}$ is defined by

27 $$ \bar{\Psi}^{\prime}(\mathbf{F}_{e},\boldsymbol{\tau})=\bar{\Psi}( \mathbf{F}_{e},\boldsymbol{\tau})-\bar{J}^{-1}\Psi _{0}( \mathbf{\bar{F}},\rho _{0},\boldsymbol{\tau}_{0}), $$

so that

28 $$ \bar{\Psi}^{\prime}(\mathbf{\bar{I}},\boldsymbol{\tau})=\bar{\Psi}( \mathbf{\bar{I}},\boldsymbol{\tau})-\bar{J}^{-1}\Psi _{0}( \mathbf{\bar{F}},\rho _{0},\boldsymbol{\tau}_{0})=0, $$

i.e. $\bar{\Psi}^{\prime}$ is measured from $\mathcal{\bar{B}}$. Equations (26) and (27) effectively define $\bar{\Psi}'$ and $\bar{\Psi}$ for given $\Psi _{0}$, $\mathbf{\bar{F}}$ and $\boldsymbol{\tau}_{0}$, for all $\mathbf{F}_{e}$ subject to $\det \mathbf{F}_{e}=1$ and $\det \mathbf{F}=\bar{J}$. The Cauchy stress connections are

29 $$ \boldsymbol{\sigma}=\bar{J}^{-1}\mathbf{F} \frac{\partial \Psi _{0}}{\partial \mathbf{F}}-p\mathbf{I}=\mathbf{F}_{e} \frac{\partial \bar{\Psi}}{\partial \mathbf{F}_{e}}-p\mathbf{I}= \mathbf{F}_{e}\frac{\partial \bar{\Psi}'}{\partial \mathbf{F}_{e}}-p \mathbf{I}. $$

### Example Free Energy Functions

For purposes of illustration in the following sections, we now consider simple examples of the free-energy functions $\Psi _{0}(\mathbf{F},\boldsymbol{\tau}_{0})$, without explicit dependence on $\rho _{0}$, and $\bar{\Psi}(\mathbf{F}_{e},\boldsymbol{\tau})$. First, we consider $\Psi _{0}$. In the transition from $\mathcal{B}_{0}$ to ℬ, $\Psi _{0}$, without any structure apart from that associated with $\boldsymbol{\tau}_{0}$, is a function of nine invariants of $\mathbf{C}$ and $\boldsymbol{\tau}_{0}$ (see, for example, [27, 28]). However, for simplicity of illustration, we consider a free energy that is a function of just three invariants, given by

30 $$ I_{1} = \operatorname{tr}\mathbf{C}, \quad I_{4}=\operatorname{tr}\boldsymbol{\tau}_{0}, \quad I_{5} = \operatorname{tr}( \boldsymbol{\tau}_{0} \mathbf{C}), $$

the first being an isotropic invariant appropriate for a material without residual stress, and the other two reflecting dependence on the residual stress. More specifically, we suppose that the dependence of $\Psi _{0}$ on these three invariants is given by

31 $$ \Psi _{0} (I_{1},I_{4},I_{5}) =\frac{1}{2}\mu \left ( I_{1} - 3 \right ) + \frac{1}{2}\left ( I_{5} - I_{4} \right ) , $$

the final term being consistent with the requirement of the dependence of the energy function on the residual stress highlighted in [27, 28], i.e. $\partial \Psi _{0}/\partial I_{5}=1/2$.

Then, by specializing (14)_1_, the Cauchy stress in ℬ is given by

32 $$ \boldsymbol{\sigma}= \bar{J}^{-1}( \mu \mathbf{B}+ \mathbf{F} \boldsymbol{\tau _{0}}\mathbf{F}^{\mathrm{T}})-p\mathbf{I}, $$

wherein the left Cauchy–Green tensor is $\mathbf{B} = \mathbf{F}\mathbf{F}^{\mathrm{T}}$ and the constant $\mu $ is the shear modulus in $\mathcal{B}_{0}$ in the absence of residual stress. When this is evaluated in $\mathcal{B}_{0}$, we obtain $p_{0}=\mu $. When it is evaluated in $\mathcal{\bar{B}}$ it gives

33 $$ \boldsymbol{\tau}=\bar{J}^{-1}( \mu \mathbf{\bar{B}}+ \mathbf{\bar{F}}\boldsymbol{\tau _{0}}\mathbf{\bar{F}}^{\mathrm{T}})- \bar{p}\mathbf{\bar{I}}, $$

with $\mathbf{\bar{B}}=\mathbf{\bar{F}}\mathbf{\bar{F}}^{\mathrm{T}}$.

As we show below, $\bar{\Psi}'$ has a similar structure to $\Psi _{0}$, namely

34 $$ \bar{\Psi}'(I_{e1},I_{e4},I_{e5})=\frac{1}{2}\bar{\mu} \left ( I_{e1} - 3 \right ) + \frac{1}{2}\left ( I_{e5} - I_{e4} \right ), $$

where

35 $$ I_{e1}=\operatorname{tr}\mathbf{C}_{e},\quad I_{e4}=\operatorname{tr}\boldsymbol{\tau}, \quad I_{e5}=\operatorname{tr}(\boldsymbol{\tau}\mathbf{C}_{e}) $$

and $\bar{\mu}$ is the shear modulus in the configuration $\mathcal{\bar{B}}$ in the absence of residual stress. This follows from the general formula (26) by considering

36 $$ \Psi _{0} (I_{1},I_{4},I_{5}) -\Psi _{0} (\bar{I}_{1},\bar{I}_{4}, \bar{I}_{5}) $$

with

37 $$ \bar{I}_{1}=\operatorname{tr}\mathbf{\bar{C}},\quad \bar{I}_{4}=\operatorname{tr} \boldsymbol{\tau}_{0},\quad \bar{I}_{5}= \operatorname{tr}( \boldsymbol{\tau}_{0} \mathbf{\bar{C}}). $$

This gives

38 $$ \frac{1}{2}\operatorname{tr}[(\mathbf{C}-\mathbf{\bar{C}})(\mu \mathbf{I}_{0}+ \boldsymbol{\tau}_{0})]=\frac{1}{2}\operatorname{tr}[(\mathbf{C}_{e}- \mathbf{\bar{I}})(\mu \mathbf{\bar{B}}+\mathbf{\bar{F}} \boldsymbol{\tau}_{0}\mathbf{\bar{F}}^{\mathrm{T}})] $$

on use of $\mathbf{\bar{C}}=\mathbf{\bar{F}}^{\mathrm{T}}\mathbf{\bar{F}}$ and $\mathbf{C}=\mathbf{\bar{F}}^{\mathrm{T}}\mathbf{C}_{e} \mathbf{\bar{F}}$. Then, by applying the expression (33) this becomes

39 $$ \frac{1}{2}\bar{J}\operatorname{tr}[(\mathbf{C}_{e}-\mathbf{\bar{I}})( \boldsymbol{\tau}+\bar{p}\mathbf{\bar{I}})]=\frac{1}{2}\bar{J}\bar{p}(I_{e1}-3) +\frac{1}{2}\bar{J}(I_{e5}-I_{e4})=\bar{J}\bar{\Psi}'. $$

Thus, from (34), $\bar{p}=\bar{\mu}$, and the Cauchy stress in (32) is also given by

40 $$ \boldsymbol{\sigma }= \bar{\mu} \mathbf{B}_{e}+ \mathbf{F}_{e} \boldsymbol{\tau}\mathbf{F}_{e}^{\mathrm{T}}-p\mathbf{I}, $$

wherein the left Cauchy–Green tensor is $\mathbf{B}_{e} = \mathbf{F}_{e} \mathbf{F}_{e}^{\mathrm{T}}$.

## Prototype Example: Growth of a Spherical Shell

To illustrate the theory, we consider its application to the problem of a growing inflated spherical shell. In the configurations $\mathcal{B}_{0}$, $\mathcal{\bar{B}}$ and ℬ, respectively, the radial coordinates of the shell are $R$, $\bar{r}$, $r$, with

41 $$ A\leq R\leq B,\quad \bar{a}\leq \bar{r}\leq \bar{b},\quad a\leq r \leq b, $$

in terms of their inner ($A$, $\bar{a}$, $a$) and outer ($B$, $\bar{b}$, $b$) radii. In each case the spherical polar angles are $\theta \in [0,\pi ]$ and $\phi \in [0,2\pi ]$.

The volume of the shell in ${\mathcal {B}}_{0}$ between $A$ and $R$ transforms (by growth) into that in $\mathcal{\bar{B}}$ between $\bar{a}$ and $\bar{r}$ and then (by elastic deformation) into the corresponding shell in ℬ between $a$ and $r$. These volumes are, respectively,

42 $$ \frac{4\pi}{3}(R^{3}-A^{3}), \quad \frac{4\pi}{3}(\bar{r}^{3}-\bar{a}^{3}), \quad \frac{4\pi}{3}(r^{3}-a^{3}). $$

We denote the volume in $\mathcal{\bar{B}}$ by $V(R)$, which is given by

43 $$ \frac{4\pi}{3}(\bar{r}^{3}-\bar{a}^{3})=V(R)=4\pi \int _{A}^{R}S^{2} \bar{J}(S)\mathrm{d}S, $$

where $\bar{J}=\bar{\lambda}_{1}\bar{\lambda}_{2}^{2}$ in terms of the stretches $\bar{\lambda}_{1}$, $\bar{\lambda}_{2}$, $S$ is a dummy variable, and the deformation $\mathbf{\bar{F}}$ has the diagonal form diag$[ \bar{\lambda}_{1}, \bar{\lambda}_{2},\bar{\lambda}_{2}]$ with

44 $$ \bar{\lambda}_{1}(R)=\mathrm{d}\bar{r}/\mathrm{d}R,\quad \bar{\lambda}_{2}(R)=\bar{r}/R. $$

Note that the volume in (43) is only equal to that in $\mathcal{B}_{0}$ when there is no growth ($\bar{J}=1$).

On the other hand, the deformation from $\mathcal{\bar{B}}$ to ℬ involves no volume change (the material is mechanically incompressible). Thus,

45 $$ r^{3}=\bar{r}^{3}+a^{3}-\bar{a}^{3},\quad \bar{r}^{3}=\bar{a}^{3}+ \frac{3}{4\pi}V(R). $$

### From $\mathcal{B}_{0}$ to $\mathcal{\bar{B}}$

Consider first the growth from $\mathcal{B}_{0}$ to $\mathcal{\bar{B}}$ in terms of the energy function $\Psi _{0}$. In this case $\rho _{0}$ depends on $\bar{J}=\bar{\lambda}_{1}\bar{\lambda}_{2}^{2}$. Thus, specialized for the considered geometry $\Psi _{0}$ depends only on the stretches $\bar{\lambda}_{1}$ and $\bar{\lambda}_{2}$ and we introduce the reduced form $\hat{\Psi}_{0}(\bar{\lambda}_{1},\bar{\lambda}_{2})=\Psi _{0}( \bar{\lambda}_{1},\bar{\lambda}_{2},\bar{\lambda}_{2})$, $\mathbf{\bar{F}}$ having the diagonal form diag$[\bar{\lambda}_{1}, \bar{\lambda}_{2},\bar{\lambda}_{2}]$ with respect to spherical polar coordinate axes, while $\boldsymbol{\tau}_{0}$ is implicit. Then, the components of the residual stress in $\mathcal{\bar{B}}$ are, on specializing (16)_1_, the radial component $\tau _{1}$ and the circumferential component $\tau _{2}$ given by

46 $$ \tau _{1}=\bar{J}^{-1}\bar{\lambda}_{1} \frac{\partial \hat{\Psi}_{0}}{\partial \bar{\lambda}_{1}}-\bar{p}, \quad \tau _{2}=\frac{1}{2}\bar{J}^{-1}\bar{\lambda}_{2} \frac{\partial \hat{\Psi}_{0}}{\partial \bar{\lambda}_{2}}-\bar{p}. $$

(Note that a Lagrange multiplier $\bar{p}$, although not used below, is included since the material is elastically incompressible.) The components $\tau _{1}$ and $\tau _{2}$ satisfy the equilibrium equation

47 $$ \frac{\mathrm{d}\tau _{1}}{\mathrm{d}\bar{r}}+ \frac{2}{\bar{r}} ( \tau _{1}-\tau _{2}) =0\quad \text{for}\ \bar{a}< \bar{r}< \bar{b}, $$

which is the appropriate specialization of (7) for the considered geometry. The corresponding boundary conditions are

48 $$ \tau _{1}=0\quad \text{for} \ \bar{r}=\bar{a},\bar{b}. $$

Integration of (47) and use of (48) yields

49 $$ \begin{aligned} \tau _{1}(\bar{r})&=\int _{\bar{a}}^{\bar{r}}\left (\bar{\lambda}_{2} \frac{\partial \hat{\Psi}_{0}}{\partial \bar{\lambda}_{2}}-2 \bar{\lambda}_{1} \frac{\partial \hat{\Psi}_{0}}{\partial \bar{\lambda}_{1}}\right ) \frac{\mathrm{d}s}{\bar{J}s},\quad \\ \mathcal{R}_{0}(\bar{a})&=\int _{ \bar{a}}^{\bar{b}}\left (\bar{\lambda}_{2} \frac{\partial \hat{\Psi}_{0}}{\partial \bar{\lambda}_{2}}-2 \bar{\lambda}_{1} \frac{\partial \hat{\Psi}_{0}}{\partial \bar{\lambda}_{1}}\right ) \frac{\mathrm{d}\bar{r}}{\bar{J}\bar{r}}=0, \end{aligned} $$

where $s$ is a dummy variable for the first integral, $\mathcal{R}_{0}(\bar{a})$ is defined by the second integral, and

50 $$ \bar{b}=\left ({\bar{a}}^{3} +\frac{3V(B)}{4\pi}\right )^{1/3}, $$

with $\tau _{2}(\bar{r})$ then obtained from (47).

Thus, when $V(R)$ is known, $\bar{r}$ is given by (45)_2_ and hence $\bar{\lambda}_{1}$ and $\bar{\lambda}_{2}$ from (44), while $V(R)$ is determined if either $\bar{\lambda}_{1}$ or $\bar{\lambda}_{2}$ is prescribed. Then, $\tau _{1}$ and $\tau _{2}$ are determined, while (49)_2_ and (50) give $\bar{a}$, and $\bar{b}$. Alternatively, if $\tau _{1}$ and $\tau _{2}$ are known, then, from (46),

51 $$ \bar{J}^{-1}\bar{\lambda}_{1} \frac{\partial \hat{\Psi}_{0}}{\partial \bar{\lambda}_{1}}- \frac{1}{2}\bar{J}^{-1}\bar{\lambda}_{2} \frac{\partial \hat{\Psi}_{0}}{\partial \bar{\lambda}_{2}}=\tau _{1}- \tau _{2} $$

is a differential equation for $\bar{r}(R)$ with $\bar{\lambda}_{1}=\mathrm{d}\bar{r}/\mathrm{d}R$, $\bar{\lambda}_{2}= \bar{r}/R$. These possible approaches will be illustrated in Sect. 4.4.

For future reference, it is worth noting that the Lagrangian counterpart of (49)_2_ can be obtained by using

52 $$ \frac{\mathrm{d}\bar{r}}{\bar{r}}= \frac{\bar{\lambda}_{1}}{\bar{\lambda}_{2}}\frac{\mathrm{d}R}{R}, $$

thus yielding

53 $$ \mathcal{R}_{0L}(\bar{a})=\int _{A}^{B}\left (\bar{\lambda}_{2} \frac{\partial \hat{\Psi}_{0}}{\partial \bar{\lambda}_{2}}-2 \bar{\lambda}_{1} \frac{\partial \hat{\Psi}_{0}}{\partial \bar{\lambda}_{1}}\right ) \frac{\bar{\lambda}_{1}}{\bar{\lambda}_{2}\bar{J}} \frac{\mathrm{d}R}{R}=0, $$

which defines $\mathcal{R}_{0L}(\bar{a})$. This is an alternative equation for $\bar{a}$.

### From $\mathcal{\bar{B}}$ to ℬ

Turning to the deformation from $\mathcal{\bar{B}}$ to ℬ, the deformation gradient $\mathbf{F}_{e}$ has the purely diagonal form diag[$\lambda ^{-2}$, $\lambda $, $\lambda $] with respect to the spherical polar axes, and the energy function $\bar{\Psi}$ depends on only one stretch, $\lambda $, and the residual stress in $\mathcal{\bar{B}}$. The components $\tau _{1}$ and $\tau _{2}$ of the residual stress are connected via (47), and $\lambda =r/\bar{r}$ can be given in the form

54 $$ \lambda =\left (a^{3} +\frac{3V(R)}{4\pi}\right )^{1/3}\bigg/\left ( \bar{a}^{3}+\frac{3V(R)}{4\pi}\right )^{1/3}. $$

In parallel with $\hat{\Psi}_{0}$, we introduce the reduced energy $\hat{\Psi}(\lambda ,\tau _{1},\tau _{2})$, which specializes the energy function $\bar{\Psi}'(\mathbf{F}_{e},\boldsymbol{\tau})$ from (27). Note that $\hat{\Psi}(1,\tau _{1},\tau _{2})=0$.

Since $V(R)$, defined in (43), depends on $\mathbf{\bar{F}}$ via $\bar{J}$, then, for a given $\mathbf{\bar{F}}$, $\lambda $ is a function of a single parameter, the inner radius $a$ of the shell in ℬ. To determine $a$ we first need to obtain an expression for the Cauchy stress in ℬ and to ensure that it satisfies the relevant equilibrium equation and boundary conditions. The Cauchy stress also has diagonal form and its principal components are denoted by $\sigma _{1}, \sigma _{2}=\sigma _{3}$.

On specialization of (23)_1_, and elimination of $p$, it follows that the radial and circumferential Cauchy stresses $\sigma _{1}$ and $\sigma _{2}$ are related by

55 $$ \sigma _{2}-\sigma _{1}=\frac{1}{2}\lambda \frac{\partial \hat{\Psi}(\lambda ,\tau _{1},\tau _{2})}{\partial \lambda}. $$

The equilibrium equation (4) for $\boldsymbol{\sigma}$ reduces to the single component equation

56 $$ \frac{\mathrm{d}\sigma _{1}}{\mathrm{d}r}+\frac{2}{r}(\sigma _{1}- \sigma _{2})=0. $$

Associated traction boundary conditions for $\sigma _{1}$ are taken to correspond to a prescribed internal pressure $P$ and no external load. Thus,

57 $$ \sigma _{1}=-P \quad \text{on}\ r=a,\quad \sigma _{1}=0 \quad \text{on}\ r=b. $$

By substituting (55) into (56), integrating with respect to $r$, using (54) and the boundary conditions (57), we obtain

58 $$ \mathcal{R}(a)=\int _{a}^{b}\lambda \frac{\partial \hat{\Psi}(\lambda ,\tau _{1},\tau _{2})}{\partial \lambda} \frac{\mathrm{d}r}{r}-P=0, $$

which defines $\mathcal{R}(a)$, where, by (45), $b$ depends on $a$:

59 $$ b=\left (a^{3} +\frac{3V(B)}{4\pi}\right )^{1/3} . $$

Thus, equation (58) together with (54) determines $a$ in terms of the growth from $\mathcal{B}_{0}$ to $\mathcal{\bar{B}}$ for a given free-energy function. Equation (58), i.e. $\mathcal{R}(a)=0$, can be solved by suitable numerical search algorithms to find $a$. Once $a$ is determined, integration of (56) yields

60 $$ \sigma _{1}(r)=\int _{a}^{r}\lambda \frac{\partial \hat{\Psi}(\lambda ,\tau _{1},\tau _{2})}{\partial \lambda} \frac{\mathrm{d}s}{s}-P. $$

Then $\sigma _{2}$ can be computed directly from (55).

Noting that equation (58) is a *spatial* description on the *unknown* current configuration ℬ, we introduce its Lagrangian counterpart, which may be more convenient for computation. From (45) and the derivative of $V(R)$ from (43), we obtain

61 $$ \frac{{\mathrm {d}}r}{r} =\frac{\bar{J}(R)}{\bar{\lambda}_{2}^{3}} \frac{{\mathrm {d}}R}{R}, $$

with $\lambda \bar{\lambda}_{2}=r/R$, so that the Lagrangian equivalent of (58) is

62 $$ \mathcal{R}_{L}(a)=\int _{A}^{B}\lambda \frac{\partial \hat{\Psi}(\lambda ,\tau _{1},\tau _{2})}{\partial \lambda} \frac{\bar{J}(R)}{\bar{\lambda}_{2}^{3}\lambda ^{3}} \frac{\mathrm{d}R}{R}-P=0. $$

This is an alternative equation for $a$.

With $a$, and hence $\lambda $, from (54), the configuration ℬ is then known, and the corresponding Cauchy stress can be computed using (60) and (55).

### Free Energy Functions

In the case of the spherical shell the free-energy functions (31) and (34) are explicitly

63 $$\begin{aligned} \hat{\Psi}_{0}(\lambda _{1},\lambda _{2}) =&\frac{1}{2}\mu \left ( \lambda _{1}^{2}+2\lambda _{2}^{2} - 3 \right ) + \frac{1}{2}\left [ \tau _{01}(\lambda _{1}^{2}-1)+2\tau _{02}(\lambda _{2}^{2}-1) \right ], \end{aligned}$$

64 $$\begin{aligned} \hat{\Psi}(\lambda ,\tau _{1},\tau _{2}) =&\frac{1}{2}\bar{\mu}\left ( \lambda ^{-4}+2\lambda ^{2} - 3 \right ) + \frac{1}{2}\left [ \tau _{1}( \lambda ^{-4}-1)+2\tau _{2}(\lambda ^{2}-1) \right ], \end{aligned}$$

in terms of the stretches $\lambda _{1}=\mathrm{d}r/\mathrm{d}R$, $\lambda _{2}=r/R$, $\lambda =r/\bar{r}$, with $\lambda $ given by (54). The components $\tau _{01}$ and $\tau _{02}$ of the residual stress in $\mathcal{B}_{0}$ will be specified shortly, while $\tau _{1}$ and $\tau _{2}$ are given by

65 $$ \tau _{1}=\bar{J}^{-1}(\mu +\tau _{01})\bar{\lambda}_{1}^{2}-\bar{p}, \quad \tau _{2}=\bar{J}^{-1}(\mu +\tau _{02})\bar{\lambda}_{2}^{2}- \bar{p}, $$

with, we recall, $\bar{\lambda}_{1}=\mathrm{d}\bar{r}/\mathrm{d}R$, $\bar{\lambda}_{2}=\bar{r}/R$ and $\bar{J}=\bar{\lambda}_{1}\bar{\lambda}_{2}^{2}$.

The components of the Cauchy stress based on $\bar{\mathcal{B}}$ are

66 $$ \sigma _{1}=(\bar{\mu}+\tau _{1})\lambda ^{-4}-p,\quad \sigma _{2}=( \bar{\mu}+\tau _{2})\lambda ^{2}-p, $$

and we note that $\bar{p}=\bar{\mu}=\mu \bar{\lambda}_{2}^{2}/\bar{J}=\mu / \bar{\lambda}_{1}$.

### Examples

#### Example 1: Isotropic Growth

First, we note that isotropic growth such that $\bar{\lambda}_{1}=\bar{\lambda}_{2}$ is not possible if there is no residual stress in $\mathcal{B}_{0}$ because then $\tau _{1}=\tau _{2}=\tau _{3}=\tau $, $\boldsymbol{\tau}=\tau \mathbf{\bar{I}}$, which leads to $\overline{\operatorname{grad}}\tau =\mathbf{0}$, and hence $\tau =$ constant, which must be zero by the boundary condition (8). See [25] for relevant background.

We therefore consider a non-trivial residual stress in $\mathcal{B}_{0}$ using the radial equilibrium and the zero traction boundary condition on $\partial \mathcal{B}_{0}$, specifically

67 $$\begin{aligned} \tau _{01} =&\alpha (R-A)(R-B)= \alpha [R^{2}-(A+B)R+AB], \\ \end{aligned}$$

68 $$\begin{aligned} \tau _{02} =&\alpha [2R^{2}-3(A+B)R/2+AB], \end{aligned}$$

analogously to that adopted for a circular cylindrical tube in [35], for example, where $\alpha >0$ is a given constant reflecting the magnitude of the residual stress.

With $\bar{\lambda}_{1}=\bar{\lambda}_{2}$ it follows from (44) that $\bar{r}=cR$, $\bar{\lambda}_{2}=c$, a constant, $\bar{J}=c^{3}$ and $\bar{p}=\mu /c$, so that from (43) it follows that

69 $$ V(R)=\frac{4\pi}{3}c^{3}(R^{3}-A^{3}). $$

Thus, $c$ is a measure of growth. From (65), (67) and (68) we obtain

70 $$ \begin{aligned} \tau _{1}(\bar{r})&=\alpha (\bar{r}-\bar{a})(\bar{r}-\bar{b}) /c^{3}= \tau _{01}/c,\quad \\ \tau _{2}(\bar{r}) &=\alpha [4\bar{r}^{2}-3(\bar{a}+ \bar{b})\bar{r}+2\bar{a}\bar{b}]/(2c^{3})=\tau _{02}/c, \end{aligned} $$

which automatically satisfies the boundary conditions (48). Note that $\bar{a}=cA$, $\bar{b}=cB$.

Using (66) in (56), we then have

71 $$ \frac{\mathrm{d}\sigma _{1}}{\mathrm{d}r}=\frac{2}{r}(\sigma _{2}- \sigma _{1})=\frac{2}{r}[(\bar{\mu}+\tau _{2})\lambda ^{2}-(\bar{\mu}+ \tau _{1})\lambda ^{-4}], $$

and more explicitly

72 $$ \frac{\mathrm{d}\sigma _{1}}{\mathrm{d}r}=2(\bar{\mu}+\bar{\alpha} \bar{a}\bar{b})\left (\frac{r}{\bar{r}^{2}}-\frac{\bar{r}^{4}}{r^{5}} \right )+2\bar{\alpha}\left (2r-\frac{\bar{r}^{6}}{r^{5}}\right )+ \bar{\alpha}(\bar{a}+\bar{b})\left (3\frac{r}{\bar{r}}-2 \frac{\bar{r}^{5}}{r^{5}}\right ) $$

where $\bar{\mu}=\mu /\bar{\lambda}_{1}=\mu /c$ and $\bar{\alpha}=\alpha /c^{3}$. Then, with $\bar{r}=(r^{3}-m^{3})^{1/3}$ and $m^{3}=a^{3}-\bar{a}^{3}$ and the help of Mathematica, we obtain

73 $$ \sigma _{1}(r)=-P+f(r)-f(a), $$

where

74 $$\begin{aligned} f(r) =&\frac{1}{2r^{4}}(\bar{\mu}+\bar{\alpha}\bar{a}\bar{b})(5r^{3}-m^{3})(r^{3}-m^{3})^{1/3}+ \frac{1}{2r^{4}}\bar{\alpha}(2r^{6}-8m^{3}r^{3}+m^{6}) \\ &{}-\frac{1}{4mr^{4}}\bar{\alpha}(\bar{a}+\bar{b})\{m^{6}H[-5/3,-4/3,-1/3,r^{3}/m^{3}] \\ &{} +3r^{6}H[1/3,2/3,5/3,r^{3}/m^{3}] \}, \end{aligned}$$

$H$ being the hypergeometric function $\text{Hypergeometric2F1}$ in Mathematica [36]. Note that since $\bar{r}>0$ by definition it follows that $r/m>1$, in which case $H$ is the analytical extension of $\text{Hypergeometric2F1}$ from the region $r/m<1$.

Then $\sigma _{2}(r)$ is obtained from (71) as

75 $$ \sigma _{2}(r)=-P+f(r)-f(a)+g(r), $$

with $g(r)$ defined by

76 $$ \begin{aligned}[b] g(r) &=(\bar{\mu}+\bar{\alpha}\bar{a}\bar{b})\frac{m^{3}}{r^{4}} \frac{(2r^{3}-m^{3})}{(r^{3}-m^{3})^{2/3}}+\frac{1}{r^{4}} \bar{\alpha}(r^{6}+2m^{3}r^{3}-m^{6}) \\ &\quad{} +\frac{1}{2r^{4}}\bar{\alpha}( \bar{a}+\bar{b}) \frac{(4m^{3}r^{3}-r^{6}+2m^{6})}{(r^{3}-m^{3})^{1/3}}. \end{aligned} $$

Since, by (59) and (69), $b^{3}=a^{3}+c^{3}(B^{3}-A^{3})$ depends on $a$ and $c$, it follows that the zero traction condition $\sigma _{1}(b)=0$ has the form

77 $$ P(a,c) =f(b)-f(a) $$

for given $\mu $, $\alpha $, $A$, $B$. This is the specialization of (58) to this example. Thus, for example, if $P$ is fixed then the inner radius $a$ increases with growth, i.e. with $c$. On the other hand, if $a$ is fixed then $P$ adjusts with growth. Note that $c>1$ for growth, while $c<1$ for atrophy.

#### Example 2: Anisotropic Growth

For anisotropic growth $\bar{\lambda}_{1}=\bar{r}'=\mathrm{d}\bar{r}/\mathrm{d}R\neq \bar{\lambda}_{2}=\bar{r}/R$ and $V(R)$ is given by (43).

We now consider the equilibrium equation (47) with (65) and $\tau _{01}=\tau _{02}=0$. Then,

78 $$ \frac{\mathrm{d}\tau _{1}}{\mathrm{d}\bar{r}}=\frac{2\mu}{\bar{r}}( \bar{\lambda}_{2}^{2}-\bar{\lambda}_{1}^{2})/\bar{J}= \frac{2\mu}{\bar{r}}(\bar{\lambda}_{1}^{-1}-\bar{\lambda}_{1} \bar{\lambda}_{2}^{-2}). $$

We take $\tau _{1}$ to have the following form, with $\tau _{2}$ obtained from the equilibrium equation:

79 $$ \tau _{1}(\bar{r})=\nu (\bar{r}-\bar{a})(\bar{r}-\bar{b}),\quad \tau _{2}( \bar{r})=\nu [4\bar{r}^{2}-3(\bar{a}+\bar{b})\bar{r}+2\bar{a}\bar{b}]/2, $$

where $\nu >0$ is a constant. By substituting (79)_1_ into (78) we obtain

80 $$ \bar{\nu}[2\bar{r}-(\bar{a}+\bar{b})]=1/(\bar{r}\bar{r}')-R^{2} \bar{r}'/\bar{r}^{3}, $$

where $\bar{\nu}=\nu /(2\mu )$. This results in a quadratic equation for $\bar{r}'$, which must be positive, and hence the relevant solution is

81 $$ \bar{r}'=\left \{-\bar{\nu} \bar{r}^{3}[2\bar{r}-(\bar{a}+\bar{b})]+ \bar{r}\left (\bar{\nu}^{2}\bar{r}^{4}[2\bar{r}-(\bar{a}+\bar{b})]^{2}+4R^{2} \right )^{1/2}\right \}\Big/(2R^{2}). $$

This determines $\bar{r}$ as a function of $R$ and hence the growth part of the deformation.

Next, from the equilibrium equation (56) with (66) we have

82 $$ \frac{\mathrm{d}\sigma _{1}}{\mathrm{d}r}=\frac{2}{r}(\sigma _{2}- \sigma _{1})=\frac{2}{r}[(\bar{\mu}+\tau _{2})\lambda ^{2}-(\bar{\mu}+ \tau _{1})\lambda ^{-4}] $$

with $\bar{\mu}=\mu /\bar{\lambda}_{1}$, $\tau _{1}$ and $\tau _{2}$ being given by (79).

Integration with respect to $r$ with $\bar{r}=(r^{3}-m^{3})^{1/3}$ and $m^{3}=a^{3}-\bar{a}^{3}$ leads to an equation of the form

83 $$ \sigma _{1}(r)=-P+k(r)-k(a), $$

where $k(r)$ results from the integration of (82) and remains to be determined numerically. Then $\sigma _{2}(r)$ is obtained from (82) as

84 $$ \sigma _{2}(r)=\sigma _{1}(r)+[(\bar{\mu}+\tau _{2})\lambda ^{2}-( \bar{\mu}+\tau _{1})\lambda ^{-4}]. $$

The boundary condition $\sigma _{1}(b)=0$ yields an equation of the form

85 $$ P=k(b)-k(a), $$

which depends on $a$, via (59) and some measure of growth to be determined from the solution of (81).

Having set up the basic framework we now consider the process of updating via the incremental quantities $\mathbf{\dot{\bar{x}}}$ (for the evolving unloaded configuration $\mathcal{\bar{B}}$) and $\boldsymbol{\dot{\tau}}$ (for the associated residual stress).

## Incremental Theory

### Incremental Equilibrium

We consider the increments, $\boldsymbol{\dot{\tau}}$ and $\mathbf{\dot{\bar{x}}}$, which are obtained from the material increment (i.e. at fixed $\mathbf{X}$). First, we take the material increment of the equilibrium equation (9), which, with $\mathbf{T}=\bar{J}\mathbf{\bar{F}}^{-1}\boldsymbol{\tau}$, gives

86 $$ \operatorname{Div}\mathbf{\dot{T}}=\operatorname{Div}\{\bar{J}\mathbf{\bar{F}}^{-1}[ \boldsymbol{\dot{\tau}} -\mathbf{\bar{L}}\boldsymbol{\tau}+\operatorname{tr}( \mathbf{\bar{L}})\boldsymbol{\tau}]\}=\mathbf{0}. $$

On pushing forward to $\mathcal{\bar{B}}$, this yields the incremental equation for $\boldsymbol{\tau}$ as

87 $$ \overline{\operatorname{div}}[\boldsymbol{\dot{\tau}} -\mathbf{\bar{L}} \boldsymbol{\tau}+\operatorname{tr}(\mathbf{\bar{L}})\boldsymbol{\tau}]= \overline{\operatorname{div}}\delta \boldsymbol{\tau}=\mathbf{0}\quad \text{in}\ \mathcal{\bar{B}}, $$

wherein the notation

88 $$ \delta \boldsymbol{\tau} = \boldsymbol{\dot{\tau}} -\mathbf{\bar{L}} \boldsymbol{\tau}+\operatorname{tr}(\mathbf{\bar{L}})\boldsymbol{\tau} $$

is introduced.

The incremental form of the boundary condition (8) is $\boldsymbol{\dot{\tau}}\mathbf{\bar{n}}+\boldsymbol{\tau} \mathbf{\dot{\bar{n}}}=\mathbf{0}$, which, on use of the standard formula $\mathbf{\dot{\bar{n}}}=[\mathbf{\bar{n}}\cdot (\mathbf{\bar{L}} \mathbf{\bar{n}})]\mathbf{\bar{n}}-\mathbf{\bar{L}}^{\mathrm{T}} \mathbf{\bar{n}}$ for the increment in $\mathbf{\bar{n}}$ and (8) itself, becomes

89 $$ (\delta \boldsymbol{\tau})\bar{\boldsymbol{n}}\equiv \boldsymbol{\dot{\tau}}\mathbf{\bar{n}}-\boldsymbol{\tau} \mathbf{\bar{L}}^{\mathrm{T}}\mathbf{\bar{n}}=\mathbf{0}\quad \text{on}\ \partial \mathcal{\bar{B}}. $$

To obtain the incremental equilibrium equation for $\boldsymbol{\sigma}$, we consider the nominal stress tensor $\mathbf{S}$ based on $\mathcal{B}_{0}$, so that $\mathbf{S}=\bar{J}\mathbf{F}^{-1}\boldsymbol{\sigma}$. On taking the increment of equation (6)_2_ we obtain the incremental equilibrium equation

90 $$ \operatorname{Div}\{\bar{J}\mathbf{F}^{-1}[\boldsymbol{\dot{\sigma}}- \mathbf{L}\boldsymbol{\sigma}+\operatorname{tr}(\mathbf{\bar{L}}) \boldsymbol{\sigma}]\}=\bar{J}\operatorname{div}[\boldsymbol{\dot{\sigma}}- \mathbf{L}\boldsymbol{\sigma}+\operatorname{tr}(\mathbf{\bar{L}}) \boldsymbol{\sigma}]. $$

Thus, the incremental equilibrium equation for $\boldsymbol{\sigma}$ is

91 $$ \operatorname{div}[\boldsymbol{\dot{\sigma}}-\mathbf{L}\boldsymbol{\sigma}+ \operatorname{tr}(\mathbf{\bar{L}})\boldsymbol{\sigma}]=\operatorname{div}\delta \boldsymbol{\sigma}=\mathbf{0}\quad \text{in}\ \mathcal{B}, $$

where, similarly to (88), we adopt the notation

92 $$ \delta \boldsymbol{\sigma}=\boldsymbol{\dot{\sigma}}-\mathbf{L} \boldsymbol{\sigma}+\operatorname{tr}(\mathbf{\bar{L}})\boldsymbol{\sigma} $$

for subsequent use.

On taking the increment of the expression (5) for the traction we obtain $\boldsymbol{\dot{\sigma}}\mathbf{n}+\boldsymbol{\sigma} \mathbf{\dot{n}}=\mathbf{\dot{t}}$ and using the formula $\mathbf{\dot{n}}=[\mathbf{n}\cdot (\mathbf{L}\mathbf{n})]\mathbf{n}- \mathbf{L}^{\mathrm{T}}\mathbf{n}$ along with (5), the following expression for the incremental Cauchy traction is obtained:

93 $$ \boldsymbol{\dot{\sigma}}\mathbf{n}=\mathbf{\dot{t}}+ \boldsymbol{\sigma}\mathbf{L}^{\mathrm{T}}\mathbf{n}-[\mathbf{n} \cdot (\mathbf{L}\mathbf{n})]\mathbf{t}. $$

Equivalently,

94 $$ (\delta \boldsymbol{\sigma})\mathbf{n}=\mathbf{\dot{t}}+[\operatorname{tr} \mathbf{L}-\mathbf{n}\cdot (\mathbf{L}\mathbf{n})]\mathbf{t}. $$

For the nominal stress based on $\mathcal{\bar{B}}$ we have $\mathbf{\bar{S}} =\mathbf{F}_{e}^{-1}\boldsymbol{\sigma}$. Then, on taking the material increment of $\mathbf{\bar{S}} =\mathbf{F}_{e}^{-1}\boldsymbol{\sigma}$, we obtain $\mathbf{\dot{\bar{S}}}=\mathbf{F}_{e}^{-1}(\boldsymbol{\dot{\sigma}}- \mathbf{L}_{e}\boldsymbol{\sigma}$), and hence, using (91),

95 $$ \overline{\operatorname{div}}[\mathbf{\dot{\bar{S}}}-\mathbf{\bar{L}} \mathbf{\bar{S}}+(\operatorname{tr}\mathbf{\bar{L}})\mathbf{\bar{S}}]= \mathbf{0}\quad \text{in}\ \mathcal{\bar{B}}. $$

This is the form of the equilibrium equation for $\mathbf{\bar{S}}$ based on $\mathcal{\bar{B}}$.

### Incremental Constitutive Law for $\boldsymbol{\tau}$ Based on $\mathcal{B}_{0}$

The constitutive laws for the Cauchy stress tensor $\boldsymbol{\sigma}$ in ℬ and the nominal stress tensor $\mathbf{S}=\bar{J}\mathbf{F}^{-1}\boldsymbol{\sigma }$ in $\mathcal{B}_{0}$ are given in equation (14). Correspondingly, the second Piola–Kirchhoff stress tensor, $\mathbf{P}=\bar{J}\mathbf{F}^{-1}\boldsymbol{\sigma }\mathbf{F}^{- \mathrm{T}}=\mathbf{S}\mathbf{F}^{-\mathrm{T}}$, in $\mathcal{B}_{0}$ is given by the constitutive law

96 $$ \mathbf{P}= \frac{\partial{{\Psi}}_{0}(\mathbf{F},\rho _{0},\boldsymbol{\tau}_{0})}{\partial \mathbf{F}} \mathbf{F}^{-\mathrm{T}}-\bar{J}p\mathbf{C}^{-1}. $$

It is convenient to obtain the incremental forms of the constitutive laws for $\boldsymbol{\sigma}$ and $\boldsymbol{\tau}$ via their counterparts for $\mathbf{P}$, as in the following.

By forming the increment of $\mathbf{P}=\bar{J}\mathbf{F}^{-1}\boldsymbol{\sigma }\mathbf{F}^{- \mathrm{T}}$ and using the relations, $\dot{\overline{\mathbf{F}^{-1}}}=-\mathbf{F}^{-1}{\mathbf{L}}$ and $\dot{\overline{\mathbf{C}^{-1}}}=-\mathbf{F}^{-1}({\mathbf{L}}+{ \mathbf{L}}^{{\mathrm{T}}})\mathbf{F}^{-{\mathrm{T}}}$, we obtain

97 $$ \dot{\mathbf{P}}=\bar{J}\mathbf{F}^{-1}[\dot{\boldsymbol{\sigma }}- \mathbf{L}\boldsymbol{\sigma }-\boldsymbol{\sigma }\mathbf{L}^{ \mathrm{T}}+(\operatorname{tr}\bar{\mathbf{L}})\boldsymbol{\sigma }]\mathbf{F}^{- \mathrm{T}}=\bar{J}\mathbf{F}^{-1} \overset{\triangledown}{\boldsymbol{\sigma }} \mathbf{F}^{-\mathrm{T}}, $$

wherein the Truesdell rate

98 $$ \overset{\triangledown}{\boldsymbol{\sigma }}= \dot{\boldsymbol{\sigma }}-\mathbf{L}\boldsymbol{\sigma }- \boldsymbol{\sigma}\mathbf{L}^{\mathrm{T}} +(\operatorname{tr}\bar{\mathbf{L}}) \boldsymbol{\sigma} $$

in ℬ is introduced. The corresponding Truesdell rate of $\boldsymbol{\tau}$ in $\bar{\mathcal{B}}$ is

99 $$ \overset{\bar{\triangledown}}{\boldsymbol{\tau}} = \dot{\boldsymbol{\tau}}-\bar{\mathbf{L}}\boldsymbol{\tau }- \boldsymbol{\tau }\bar{\mathbf{L}}^{\mathrm{T}} +(\operatorname{tr} \bar{\mathbf{L}})\boldsymbol{\tau}. $$

Next, by taking the increment of (96) we obtain

100 $$ \dot{\mathbf{P}}=\bar{J}\mathbf{F}^{-1}\left \{\bar{J}^{-1}\left [ \mathbf{F} \frac{\partial ^{2}{\Psi}_{0}}{\partial \mathbf{F}\partial \mathbf{F}} \mathbf{F}^{\mathrm{T}}\right ] :\mathbf{L}-\boldsymbol{\sigma }{ \mathbf{L}}^{\mathrm{T}}+p[{\mathbf{L}}-(\operatorname{tr}\mathbf{\bar{L}}) \mathbf{I}]- \dot{p}\mathbf{I}\right \}\mathbf{F}^{-\mathrm{T}}, $$

and hence, from (97),

101 $$ \begin{aligned}[b] \overset{\triangledown}{\boldsymbol{\sigma }} &= \bar{J}^{-1}\left [ \mathbf{F} \frac{\partial ^{2}{\Psi}_{0}}{\partial \mathbf{F}\partial \mathbf{F}} \mathbf{F}^{\mathrm{T}}\right ]:\mathbf{L} -\boldsymbol{\sigma }{ \mathbf{L}}^{\mathrm{T}}+ p[{\mathbf{L}}-(\operatorname{tr}\mathbf{\bar{L}}) \mathbf{I}]-\dot{p}\mathbf{I} \\ & \equiv \dot{\boldsymbol{\sigma}}- \mathbf{L}\boldsymbol{\sigma} -\boldsymbol{\sigma }{\mathbf{L}}^{ \mathrm{T}}+(\operatorname{tr}\bar{\mathbf{L}})\boldsymbol{\sigma}. \end{aligned} $$

For convenience, equation (101) may be re-expressed as

102 $$ \delta{\boldsymbol{\sigma }}= \bar{J}^{-1}\left [\mathbf{F} \frac{\partial ^{2}{\Psi}_{0}}{\partial \mathbf{F}\partial \mathbf{F}} \mathbf{F}^{\mathrm{T}}\right ]:\mathbf{L}-\delta \mathbf{p}, $$

where

103 $$ \delta \mathbf{p}=\dot{p}\mathbf{I}-p[{\mathbf{L}}-(\operatorname{tr} \mathbf{\bar{L}}) \mathbf{I}]. $$

Similarly, we may also write

104 $$ \delta \boldsymbol{\tau}= \bar{J}^{-1}\left [\bar{\mathbf{F}} \frac{\partial ^{2}{\Psi}_{0}({\mathbf{F}},\rho _{0},\boldsymbol{\tau}_{0})}{\partial \mathbf{F}\partial \mathbf{F}} \bigg\vert _{\mathbf{F}=\bar{\mathbf{F}}} \bar{\mathbf{F}}^{ \mathrm{T}}\right ]:\bar{\mathbf{L}}-\delta \bar {\mathbf{p}}, $$

where

105 $$ \delta \bar {\mathbf{p}}= \dot {\bar{p}}\bar{\mathbf{I}}-\bar{p}[ \bar{\mathbf{L}} -(\operatorname{tr}\mathbf{\bar{L}}) \bar{\mathbf{I}}] . $$

### A Specific Incremental Constitutive Law for $\boldsymbol{\tau}$

We now apply the above theory to the specific constitutive law for ${\Psi}_{0}$ given in equation (31) with the invariants defined in (30). We first calculate, in components,

106 $$ \frac{\partial{\Psi}_{0}}{\partial F_{i\alpha}} =~\upmu \text{F}_{i\alpha} + \boldsymbol{\tau}_{0\alpha \gamma}F_{i\gamma},\quad \frac{\partial ^{2}\Psi _{0}}{\partial F_{i\alpha}\partial F_{j\beta}}= \mu \delta _{ij}\delta _{\alpha \beta}+\delta _{ij}\tau _{0\alpha \beta}. $$

Hence

107 $$ \left (\mathbf{F} \frac{\partial ^{2}\Psi _{0}}{\partial \mathbf{F}\partial \mathbf{F}} \mathbf{F}^{\mathrm{T}}\right )_{piqj}=\mu \delta _{ij}B_{pq}+\delta _{ij} \boldsymbol{\Sigma}_{0pq}, $$

where $B_{pq}$ are the components of the left Cauchy–Green tensor $\mathbf{B}=\mathbf{F}\mathbf{F}^{\mathrm{T}}$ and $\boldsymbol{\Sigma}_{0}=\mathbf{F}\boldsymbol{\tau}_{0}\mathbf{F}^{ \mathrm{T}}$. This can then be specialized for $\mathbf{F}=\bar{\mathbf{F}}$ as required in (104).

It follows that

108 $$ \left (\mathbf{F} \frac{\partial ^{2}\Psi _{0}}{\partial \mathbf{F}\partial \mathbf{F}} \mathbf{F}^{\mathrm{T}}\right ):\mathbf{L}=\mu \mathbf{LB}+\mathbf{L} \boldsymbol{\Sigma}_{0} $$

### Stress Driven Growth

The residual stress $\boldsymbol{\tau}$ evolves with growth, and growth is in part driven by the application of loads and, within ℬ, the Cauchy stress $\boldsymbol{\sigma}$ thereby produced. Thus, $\boldsymbol{\tau}$ can be considered as a measure of growth, and it changes with growth, as in the example of the opening angle experiment (see [37]), which can be used to measure the opening angle at different stages of growth.

It is therefore appropriate to consider changes of $\boldsymbol{\tau}$ to depend on $\boldsymbol{\tau}$ itself and on $\boldsymbol{\sigma}$. Such a dependence can be written in the fairly general form

109 $$ \boldsymbol{\dot{\tau}}=\boldsymbol{\mathcal{G}}(\boldsymbol{\tau}, \boldsymbol{\sigma}) , $$

where $\boldsymbol{\dot{\tau}}$ is the increment in $\boldsymbol{\tau}$ and $\boldsymbol{\mathcal{G}}$ is a symmetric tensor function. Even more generally, an implicit relation of the form $\mathcal{G}(\boldsymbol{\dot{\tau}},\boldsymbol{\tau}, \boldsymbol{\sigma})=0$ could be considered, but here it is sufficient to focus on (109) as the growth law applying in the evolving region $\mathcal{\bar{B}}$. The function $\boldsymbol{\mathcal{G}}$ will be specialized for specific problems subsequently.

By using equation (104) with (88) and (105), we obtain

$$ \boldsymbol{\dot{\tau}} = \bar{J}^{-1}\left [\bar{\mathbf{F}} \frac{\partial ^{2}{\Psi}_{0}({\mathbf{F}},\rho _{0},\boldsymbol{\tau}_{0})}{\partial \mathbf{F}\partial \mathbf{F}} \bigg\vert _{\mathbf{F}=\bar{\mathbf{F}}}\bar{\mathbf{F}}^{\mathrm{T}} \right ]:\bar{\mathbf{L}} - \dot {\bar{p}}\bar{\mathbf{I}} + \mathbf{\bar{L}}(\boldsymbol{\tau} + \bar{p}\bar{\mathbf{I}}) - ( \operatorname{tr}\mathbf{\bar{L}})(\boldsymbol{\tau} + \bar{p}\bar{\mathbf{I}}). $$

Then, by eliminating $\dot{\bar{p}}$ from the diagonal terms (no summation of $i$ or $j$), we have

110 $$\begin{aligned} \dot{\tau}_{ii}-\dot{\tau}_{jj} =& \left [\bar{J}^{-1}\left [ \bar{\mathbf{F}} \frac{\partial ^{2}{\Psi}_{0}({\mathbf{F}},\rho _{0},\boldsymbol{\tau}_{0})}{\partial \mathbf{F}\partial \mathbf{F}} \bigg\vert _{\mathbf{F} = \bar{\mathbf{F}}}\bar{\mathbf{F}}^{ \mathrm{T}}\right ]:\bar{\mathbf{L}}\right ]_{ii} \\ &{} - \left [\bar{J}^{-1} \left [\bar{\mathbf{F}} \frac{\partial ^{2}{\Psi}_{0}({\mathbf{F}},\rho _{0},\boldsymbol{\tau}_{0})}{\partial \mathbf{F}\partial \mathbf{F}} \bigg\vert _{\mathbf{F} = \bar{\mathbf{F}}}\bar{\mathbf{F}}^{ \mathrm{T}}\right ]:\bar{\mathbf{L}}\right ]_{jj} \\ &{} +\left [\mathbf{\bar{L}}(\boldsymbol{\tau}+\bar{p}\bar{\mathbf{I}}) \right ]_{ii} - \left [\mathbf{\bar{L}}(\boldsymbol{\tau}+\bar{p} \bar{\mathbf{I}})\right ]_{jj} - (\operatorname{tr}\mathbf{\bar{L}})({\tau _{ii}- \tau _{jj}}). \end{aligned}$$

The non-diagonal terms ($i\neq j$) are

111 $$ \dot{\tau}_{ij} = \left [\bar{J}^{-1}\left [\bar{\mathbf{F}} \frac{\partial ^{2}{\Psi}_{0}({\mathbf{F}},\rho _{0},\boldsymbol{\tau}_{0})}{\partial \mathbf{F}\partial \mathbf{F}} \bigg\vert _{\mathbf{F}=\bar{\mathbf{F}}}\bar{\mathbf{F}}^{\mathrm{T}} \right ]:\bar{\mathbf{L}}\right ]_{ij} +\left [\mathbf{\bar{L}}( \boldsymbol{\tau}+\bar{p}\bar{\mathbf{I}})\right ]_{ij} - (\operatorname{tr} \mathbf{\bar{L}}){\tau}_{ij}. $$

Therefore, once $\boldsymbol{\dot{\tau}}$ is specified by the function $\boldsymbol{\mathcal{G}}$ in (109), equatiions (110) and (111) may be taken as the equations to determine $\bar{\mathbf{L}}$. Based on this observation, we may consider an alternative growth law,

112 $$ \bar{\mathbf{L}}=\boldsymbol{\hat{\mathcal{G}}}(\boldsymbol{\tau}, \boldsymbol{\sigma}) , $$

where $\boldsymbol{\hat{\mathcal{G}}}$ is a tensor function. Note that $\bar{\mathbf{L}}$ is a *compatible* ‘velocity’ gradient.

## General Programme

We now outline one possible general procedure for obtaining the evolving $\mathcal{\bar{B}}$ and $\boldsymbol{\tau}$, without specifying any particular form of free-energy function. Given $\mathcal{\bar{B}}$ and $\boldsymbol{\tau}$ at time $t$, the following steps are followed.

1. Step 1:
  With the energy function $\bar{\Psi}$ based on the configuration $\mathcal{\bar{B}}$, obtain the Cauchy stress $\boldsymbol{\sigma}$ in ℬ given by (23)_1_ as a function of $\mathbf{F}_{e}=\overline{\operatorname{grad}}\mathbf{x}$ and $\boldsymbol{\tau}$, eliminate $p$ using the equation $\text{curl}(\operatorname{div}\boldsymbol{\sigma})=\mathbf{0}$. Solve this equation in the pulled-back form $\overline{\text{curl}}(\mathbf{F}_{e}^{\mathrm{T}} \overline{\operatorname{div}}\mathbf{\bar{S}})=\mathbf{0}$ with $\mathbf{\bar{S}}=\mathbf{F}_{e}^{-1}\boldsymbol{\sigma}$ from (23)_2_ to obtain $\mathbf{x}=\boldsymbol{\chi}_{e}(\mathbf{\bar{x}})$. Then obtain $\overline{\operatorname{grad}}p$, and hence $p$, from $\overline{\operatorname{div}}\mathbf{\bar{S}}=\mathbf{0}$. Hence obtain $\mathbf{\bar{S}}$ from (23)_2_ and $\boldsymbol{\sigma}$ from (23)_1_ subject to appropriate boundary conditions on $\partial \mathcal{B}$. This yields both ℬ and $\boldsymbol{\sigma}$ at time $t$. Use the information to check if growth stops; if not, continue the iteration as required.
2. Step 2:
  Next, form the increment $\boldsymbol{\dot{\tau}}$ of $\boldsymbol{\tau}$ from (109). Form equations (110) and (111) to eliminate $\dot{\bar{p}}$ and solve $\bar{\mathbf{L}}=\overline{\operatorname{grad}}\mathbf{\dot{\bar{x}}}$ for $\mathbf{\dot{\bar{x}}}$. Then obtain $\dot{\bar{p}}$ from equation (104). This yields both $\mathbf{\dot{\bar{x}}}$ and $\boldsymbol{\dot{\tau}}$, which is subject to the zero (incremental) traction boundary condition (89), and hence the updated $\mathcal{\bar{B}}$ and $\boldsymbol{\tau}$ for a new time step $t+\Delta t$.
3. Step 3:
  With $\mathcal{\bar{B}}$ and $\boldsymbol{\tau}$ updated, we may go to Step 1 to update ℬ and $\boldsymbol{\sigma}$ for the new time step $t+\Delta t$. Alternatively, we may compute the increments of ℬ and $\boldsymbol{\sigma}$ as follows.
  Form the increment $\mathbf{\dot{\bar{S}}}$ from (23)_2_ with $\mathbf{\dot{F}}_{e}=\overline{\operatorname{grad}}\mathbf{\dot{x}}$. This satisfies the incremental equilibrium equation (95). Eliminate $\dot{p}$ by taking the $\overline{\text{curl}}$ of this equation, and solve the resulting equation for $\mathbf{\dot{x}}$. Then obtain $\overline{\operatorname{grad}}\dot{p}$, and hence $\dot{p}$, from the equation (95) subject to suitable (incremental) boundary conditions. This yields both $\mathbf{\dot{x}}$ and $\mathbf{\dot{\bar{S}}}$, hence the updated ℬ, while $\boldsymbol{\sigma}$ is updated to $\boldsymbol{\dot{\sigma}}$ from the connection $\boldsymbol{\dot{\sigma}}=\mathbf{F}_{e}\mathbf{\dot{\bar{S}}}+ \mathbf{L}_{e}\boldsymbol{\sigma}$.
  Now return to Step 2, use the updated $\boldsymbol{\tau}$ and $\boldsymbol{\sigma}$ to further update $\boldsymbol{\dot{\tau}}$ from the growth law (109) to continue the iteration as required until growth stops (i.e. when $\boldsymbol{\dot{\tau}}=\mathbf{0}$).

This general approach works in two or three dimensions, when $p$ and $\bar{p}$ can be eliminated initially. However, for a one-dimensional problem $p$ and $\bar{p}$ cannot be eliminated in the same way, so a different procedure is then required, as illustrated for the radial deformation of a spherical shell in the following.

## Incremental Growth of a Spherical Shell

### Equilibrium Equations

For this problem the incremental quantities relating $\mathcal{\bar{B}}$ to $\mathcal{B}_{0}$ have the forms $\boldsymbol{\dot{\tau}} =\operatorname{diag} [\dot{\tau}_{1}, \dot{\tau}_{2}, \dot{\tau}_{2}]$, $\mathbf{\dot{\bar{x}}}=(\dot{\bar{r}},0,0)$, $\mathbf{\bar{L}}=\operatorname{diag}[\bar{L}_{11},\bar{L}_{22},\bar{L}_{22}]= \operatorname{diag}[\mathrm{d}\dot{\bar{r}}/\mathrm{d}\bar{r},\dot{\bar{r}}/ \bar{r},\dot{\bar{r}}/\bar{r}]$ with respect to spherical polar axes, while $\mathbf{\bar{n}}=(-1,0,0)$ on $\bar{r}=\bar{a}$, $\mathbf{\bar{n}}=(1,0,0)$ on $\bar{r}=\bar{b}$.

Equation (87) specializes to

113 $$ \frac{\mathrm{d}}{\mathrm{d}\bar{r}}(\delta \tau _{1})+ \frac{2}{\bar{r}}(\delta \tau _{1}-\delta \tau _{2})=0\quad \text{in} \ \bar{a}< \bar{r}< \bar{b}, $$

while the boundary condition (89) reduces

114 $$ \delta \tau _{1}=0\quad \text{on}\ \bar{r}=\bar{a}, \bar{b}. $$

Or equivalently we write equation (113) and boundary condition (114) as

115 $$ \frac{\mathrm{d}\dot{\tau}_{1}}{\mathrm{d}\bar{r}}+\frac{2}{\bar{r}}( \dot{\tau}_{1}-\dot{\tau}_{2})+\frac{2}{\bar{r}}(\tau _{1}-\tau _{2})( \bar{L}_{11}-\bar{L}_{22})=0\quad \text{in} \ \bar{a}< \bar{r}< \bar{b}, $$

and

116 $$ \dot{\tau}_{1}=0\quad \text{on}\ \bar{r}=\bar{a}, \bar{b}. $$

Integration of (113) and use of the boundary conditions (114) leads to

117 $$ \delta \tau _{1}=2\int _{\bar{a}}^{\bar{r}}(\delta \tau _{1}-\delta \tau _{2})\frac{\mathrm{d}\bar{r}}{\bar{r}}, $$

and

118 $$ \int _{\bar{a}}^{\bar{b}}(\delta \tau _{1}-\delta \tau _{2}) \frac{\mathrm{d}\bar{r}}{\bar{r}}=0, $$

$\delta \tau _{2}$ then being obtained from (113).

For the incremental quantities relating ℬ to $\mathcal{\bar{B}}$, on taking the increment of (45)_1_, we obtain

119 $$ r^{2}\dot{r}=\bar{r}^{2}\dot{\bar{r}}+a^{2}\dot{a}-\bar{a}^{2} \dot{\bar{a}}. $$

Then, the increment of $\lambda =r/\bar{r}$ gives

120 $$ \dot{\lambda}=\dot{r}/\bar{r}-r\dot{\bar{r}}\bar{r}^{-2}, $$

which, using (119) and (45)_1_, can be reorganized as

121 $$ \dot{\lambda}= \frac{[(\bar{a}^{3}-a^{3})\dot{\bar{r}}+(a^{2}\dot{a}-\bar{a}^{2}\dot{\bar{a}})\bar{r}]}{\bar{r}^{2}(\bar{r}^{3}+a^{3}-\bar{a}^{3})^{2/3}}, $$

this depending on quantities associated with the transition from $\mathcal{B}_{0}$ to $\mathcal{\bar{B}}$ to ℬ.

From (91), we obtain the incremental equilibrium equation

122 $$ \frac{\mathrm{d}}{\mathrm{d}r}(\delta \sigma _{1})+\frac{2}{r}( \delta \sigma _{1}-\delta \sigma _{2})=0\quad \text{in} \ a< r< b. $$

The corresponding incremental boundary conditions are obtained by specializing (94) to give

123 $$ \delta \sigma _{1}=-\dot{P}-2PL_{22}\quad \text{on}\ r=a,\quad \delta \sigma _{1}= 0 \quad \text{on} \ r=b, $$

recalling that $L_{22}=\dot{r}/r$.

Integration of (122) and application of the boundary conditions (123) leads to

124 $$ \delta \sigma _{1}+2\int _{a}^{r}(\delta \sigma _{1}-\delta \sigma _{2}) \frac{\mathrm{d}r}{r}+\dot{P}+2PL_{22}\vert _{r=a}=0, $$

125 $$ \dot{P}=2\int _{a}^{b}(\delta \sigma _{2}-\delta \sigma _{1}) \frac{\mathrm{d}r}{r}-2PL_{22}\vert _{r=a}. $$

In (124) $\delta \sigma _{1}-\delta \sigma _{2}$ will be given for a specific constitutive law in what follows in terms of $\lambda $, $\tau _{1}$, $\tau _{2}$ and their increments, while (125) gives $\dot{P}$ in terms of $\dot{a}$ and quantities known in $\bar{\mathcal{B}}$.

### A Specific Incremental Constitutive Law

Since $\mathbf{L}$, $\mathbf{B}$ and $\boldsymbol{\Sigma}_{0}$ are diagonal with respect to the spherical polar axes for the considered spherical shell the free-energy function (31) is given explicitly in (63) in terms of the stretches $\lambda _{1}=\mathrm{d}r/\mathrm{d}R$, $\lambda _{2}=r/R$. It follows that (108) is purely diagonal and hence equation (102) has the diagonal components

126 $$ \delta \sigma _{1}= \bar{J}^{-1}\lambda _{1}^{2}(\mu +\tau _{01}){L}_{11}- \delta{p}_{1},\quad \delta \sigma _{2}=\bar{J}^{-1}\lambda _{2}^{2}( \mu +\tau _{02}){L}_{22}-\delta{p}_{2}, $$

where, bearing in mind that $\operatorname{tr}\mathbf{L}=\operatorname{tr}\bar{\mathbf{L}}$,

127 $$ \delta{p}_{1}= \dot{p} +2pL_{22},\quad \delta{p}_{2}=\dot{p} +p(L_{11}+L_{22}), $$

and $L_{11}=\dot{\lambda}_{1}/\lambda _{1}$, $L_{22}=\dot{\lambda}_{2}/\lambda _{2}$.

Similarly

128 $$ \delta \tau _{1}=\bar{J}^{-1}\bar{\lambda}_{1}^{2}(\mu +\tau _{01}) \bar{L}_{11}-\delta \bar{p}_{1},\quad \delta \tau _{2}=\bar{J}^{-1} \bar{\lambda}_{2}^{2}(\mu +\tau _{02})\bar{L}_{22}-\delta \bar{p}_{2}, $$

where

129 $$ \delta \bar{p}_{1}= \dot{\bar{p}}+2\bar{p}\bar{L}_{22},\quad \delta \bar{p}_{2}= \dot{\bar{p}}+\bar{p}(\bar{L}_{11}+\bar{L}_{22}), $$

and $\bar{L}_{11}=\dot{\bar{\lambda}}_{1}/\bar{\lambda}_{1}$, $\bar{L}_{22}=\dot{\bar{\lambda}}_{2}/\bar{\lambda}_{2}$.

The differences $\delta \sigma _{1}-\delta \sigma _{2}$ and $\delta \tau _{1}-\delta \tau _{2}$ are to be used in equations (124) and (117), respectively.

Substituting equation (128) into equation (117) gives the relation

130 $$ \delta \tau _{1}-\delta \tau _{2} = \gamma _{01}\bar{{L}}_{11} - \gamma _{02}\bar{{L}}_{22}, $$

where

$$ \gamma _{01}=\bar{J}^{-1}\bar{\lambda}_{1}^{2}(\mu +\tau _{01}) + \bar{p},\quad \gamma _{02}=\bar{J}^{-1}\bar{\lambda}_{2}^{2}(\mu + \tau _{02})+\bar{p}. $$

Then, substituting equation (130) in to right-hand side of equation (113) and integrating the equation obtained yields

131 $$ \delta \tau _{1}(\bar{r})=-\int _{\bar{a}}^{\bar{r}}\mathcal{H}_{\tau}(s) \,\mathrm{d}{s} $$

subject to the traction-free boundary condition

132 $$ \delta \tau _{1}(\bar{b})=-\int _{\bar{a}}^{\bar{b}}\mathcal{H}_{\tau}( \bar{r}),\mathrm{d}{\bar{r}}=0, $$

where the function $\mathcal{H}_{\tau}$ is defined as

133 $$ \mathcal{H}_{\tau}(\bar{r})=\frac{2}{\bar{r}}(\gamma _{01}\bar{{L}}_{11} - \gamma _{02}\bar{{L}}_{22}). $$

Consequently, $\delta \tau _{2}$ is computed from equation (130) as

134 $$ \delta \tau _{2}(\bar{r})=\delta \tau _{1}(\bar{r})-\frac{\bar{r}}{2} \mathcal{H}_{\tau}(\bar {r}). $$

### Possible Growth Laws for the Spherical Shell

As mentioned above in the general programme, both the evolving residual stress and the configuration $\mathcal{\bar{B}}$ can in principle be determined, but the programme does not apply to the case of the spherical shell. Thus, we now consider five possible forms of the growth law as applicable to the spherical shell. Classes 1–3 are aligned with (109) while Classes 4, 5 with (112).

#### Class 1: $\delta \boldsymbol{{\tau}}$ Is Prescribed

Given the components $(\delta \tau _{1},\delta \tau _{2})$ of $\delta \boldsymbol{{\tau}}$, $\dot{\bar{r}}$ can be obtained by integration of (130), along with $\mathbf{\bar{L}}=\operatorname{diag}[\bar{L}_{11},\bar{L}_{22},\bar{L}_{22}]= \operatorname{diag}[\mathrm{d}\dot{\bar{r}}/\mathrm{d}\bar{r},\dot{\bar{r}}/ \bar{r},\dot{\bar{r}}/\bar{r}]$ and requires that the boundary value $\dot{\bar{a}}$ (or $\dot{\bar{b}}$) is known.

#### Class 2: ${\delta \tau _{2}}$ Is Prescribed

As distinct from Case 1, $\delta \tau _{1}$ can be determined solely from the equilibrium equation (113). Then, with both $\delta \tau _{1}$ and $\delta \tau _{2}$ known, $\mathbf{\bar{L}}$ can be determined by using the constitutive law (128) and the compatibility condition of $\mathbf{\bar{L}}$, i.e. $\bar{L}_{11}=\mathrm{d}(\bar{r}\bar{L}_{22})/\mathrm{d}\bar{r}$. Again, the boundary value $\dot{\bar{a}}$ (or $\dot{\bar{b}}$) is required.

#### Class 3: ${\dot{\tau}_{2}}$ Is Prescribed

In this case $\dot{\tau}_{2}$ is known, and we need to integrate the fully coupled set of equations including the equilibrium equation (113), the constitutive law (128), and the compatibility condition for $\mathbf{\bar{L}}$ to determine $\dot{\tau}_{1}$ and $\mathbf{\bar{L}}$. The boundary value $\dot{\bar{a}}$ (or $\dot{\bar{b}}$) is also required.

#### Class 4: $\dot{\bar{r}}$ Is Prescribed

Given $\dot{\bar{r}}$, $\delta \tau _{1}-\delta \tau _{2}$ can be determined from the constitutive law (128). Then, substituting the expression of $\delta \tau _{1}-\delta \tau _{2}$ into the equilibrium equation (113) and integrating will determine $\delta \tau _{1}$, and $\delta \tau _{2}$ can then be obtained from $\delta \tau _{1}-\delta \tau _{2}$ and (128).

#### Class 5: $\operatorname{tr}\mathbf{\bar{L}}$ Is Prescribed

With $\operatorname{tr}\mathbf{\bar{L}}$ known, $\dot {\bar{r}}$ can be obtained by integration (with $\dot{\bar{a}}$ (or $\dot{\bar{b}}$) undetermined) and hence $\mathbf{\bar{L}}$, which gives $\delta \tau _{1}-\delta \tau _{2}$ via the constitutive law (128). Consequently $\delta \tau _{1}$ can be integrated from the equilibrium equation (113). In turn, the boundary condition $\delta \tau _{1}=0$ at $R=A$ provides the condition for determining $\dot{\bar{a}}$ (or $\dot{\bar{b}}$).

As an example, we apply Class 1 to the spherical shell in the following.

### A Procedure for the Spherical Shell

The components $(\delta \tau _{1},\delta \tau _{2})$ of $\delta \boldsymbol{{\tau}}$ at time $t$ are given by a growth law.

1. Step 1:
  Integrate (56) with (55) and the boundary conditions (57) to obtain the dependence of $a$ on $P$, as in (58). From (60) and (55) this yields $\sigma _{1}$, $\sigma _{2}$, as well as $r$, and hence ℬ at time $t$. Use the information to check if growth stops, if not, continue the iteration as required.
2. Step 2:
  Form the increments $(\delta \tau _{1},\delta \tau _{2})$ from a growth law. Form equation (130) to eliminate $\dot{\bar{p}}$ and solve $\operatorname{diag}[\bar{L}_{11},\bar{L}_{22}]=\operatorname{diag}[\mathrm{d} \dot{\bar{r}}/\mathrm{d}\bar{r},\dot{\bar{r}}/\bar{r}]$ for $\dot{\bar{r}}(R)$. Then obtain $\dot{\bar{p}}$ from equation (128). This yields $\dot{\bar{r}}$, $\delta \tau _{1}$, $\delta \tau _{2}$ and hence the updated $\mathcal{\bar{B}}$ for a new time step $t+\Delta t$.
3. Step 3:
  With $\mathcal{\bar{B}}$ and $\boldsymbol{\tau}$ updated, we may go to Step 1 to update ℬ and $\boldsymbol{\sigma}$ for the new time step $t+\Delta t$ and continue the iteration as required until growth stops (i.e. when $\delta \tau _{1}=\delta \tau _{2}=0$ and $\dot{\bar{r}}=0$). Alternatively, we may compute the increments of ℬ and $\boldsymbol{\sigma}$ as follows.
  With the expressions for $\dot{r}$ and $\dot{\lambda}$ from (119) and (121), which depend on $\dot{a}$, integrate equation (122) with the boundary conditions (123) and hence obtain the relationship (125) between $\dot{a}$ and $\dot{P}$. The expressions for $\delta \sigma _{1}$ and $\delta \sigma _{2}$ follow from (124) and (122). This yields $\dot{r}$, $\dot{\sigma}_{1}$ and $\dot{\sigma}_{2}$, and hence the updated ℬ for the new time step $t+\Delta t$. With the updated $\tau _{1}$, $\tau _{2}$, $\sigma _{1}$ and $\sigma _{2}$ return to Step 2 to continue the iteration until growth stops.

## Application: Growth of an Incompressible Elastic Spherical Shell Regulated by Homeostasis

For application purposes, we consider that the growth is regulated by homeostasis as a prototype for demonstrating the proposed method. Growth regulated by homeostasis has been widely illustrated in literature [31]. Homeostasis plays the role of a mechanism that drives the growth to a specific steady state. One of the well-known examples of a homeostatic state is the uniform distribution of circumferential stress through the radial direction of a cylindrical artery wall, which is related to the residual stress in the wall manifested by an opening angle when the wall is cut along the radical direction [8, 9].

As an example, we assume such a homeostatic condition in the spherical shell,

135 $$ \sigma _{2}(r)\big|_{t\to \infty}=\sigma _{3}(r)\big|_{t\to \infty}= \sigma _{h}\quad \forall r\in [a,b], $$

where $\sigma _{h}$ is a circumferential homeostatic stress. We shall discuss the formulation of growth regulated by the homeostatic condition (135) for computing the increments of $\boldsymbol{\tau}$ and $\bar{\mathcal{B}}$ using the proposed five classes of the growth laws.

### Class 1: Specifications of $\delta \boldsymbol{{\tau}}$ and $\dot{\bar{a}}$

Considering objectivity and measurability, the general growth law (109)_1_ may be specified in the slightly different form

136 $$ \delta{\boldsymbol{\tau }} = \mathcal{G}^{*}\left (\boldsymbol{\tau}, \boldsymbol{\sigma}\right ). $$

One of the components of $\delta \boldsymbol{\tau}$, say $\delta \tau _{1}$, may be assumed to have the following form, with the other component, $\delta \tau _{2}$, obtained from the equilibrium equation (113):

137 $$ \delta \tau _{1}(\bar{r})=\delta \nu (\bar{r}-\bar{a})(\bar{r}- \bar{b}),\quad \delta \tau _{2}(\bar{r}) =\delta \nu [4\bar{r}^{2}-3( \bar{a}+\bar{b})\bar{r}+2\bar{a}\bar{b}]/2, $$

where $\delta \nu >0$ is a constant parameter across $\bar{a}\leq \bar{r}\leq \bar{b}$. Equation (137) is analogous to the flow rule in viscoplasticity theory. Hence, the parameter $\delta \nu $ may be taken as

138 $$ \delta \nu = -{\eta _{\nu}}(\bar{\sigma}_{2}-\sigma _{h}), $$

where $\eta _{\nu}$ is a model parameter, and

139 $$ \bar{\sigma}_{2}=\frac{2}{b^{2}-a^{2}}\int _{a}^{b}\sigma _{2}(r)r \mathrm{d}r $$

is the average value of $\sigma _{2}$. Note that, different from the local flow rule in viscoplasticity theory, the parameter $\delta \nu $ and (137) are non-local.

By substituting (137)_1_ and (130) into the first and second terms in the equilibrium equation (113), respectively, we obtain

140 $$ \delta \nu [2\bar{r}-(\bar{a}+\bar{b})] + \frac{2}{\bar{r}}(\gamma _{01} \bar{{L}}_{11} -\gamma _{02}\bar{{L}}_{22})=0. $$

Using $\mathbf{\bar{L}}=\operatorname{diag}[\bar{L}_{11},\bar{L}_{22},\bar{L}_{22}]= \operatorname{diag}[\mathrm{d}\dot{\bar{r}}/\mathrm{d}\bar{r},\dot{\bar{r}}/ \bar{r},\dot{\bar{r}}/\bar{r}]$, the above equation becomes an ordinary differential equation for $\dot{\bar{r}}$:

141 $$ \gamma _{01}\frac{\mathrm{d}\dot{\bar{r}}}{\mathrm{d}\bar{r}} - \gamma _{02}\frac{\dot{\bar{r}}}{\bar{r}} + \delta \nu \frac{\bar{r}[2\bar{r}-(\bar{a}+\bar{b})]}{2} = 0. $$

Equation (141) determines $\dot{\bar{r}}$ as a function of $\bar{r}$ given the boundary condition $\dot{\bar{a}}$ (or $\dot{\bar{b}}$) as a separate growth law (see Sect. 8.6.2). In principle $\dot{\bar{r}}$ at boundaries $R=A, B$ is measurable, which could provide a physically realistic boundary condition.

Consequently, $\dot{\bar{p}}$ can be computed using equation (128) as

142 $$ \dot{\bar{p}} = \gamma _{01}\bar{L}_{11} - \delta \tau _{1}-\bar{p} \operatorname{tr}\mathbf{\bar{L}}\quad{\mathrm{or}}\quad \dot{\bar{p}} = \gamma _{02}\bar{L}_{22} - \delta \tau _{2}-\bar{p}\operatorname{tr} \mathbf{\bar{L}}. $$

This completes the computation of the increments of $\boldsymbol{\tau}$ and $\bar{\mathcal{B}}$.

Note that equations (137) and (138) only lead to a uniform spatial distribution of $\sigma _{2}$ which is not necessarily equal to $\sigma _{h}$ since the expression for $\delta \tau _{2}$ in (137)_2_ does not include an arbitrary constant term. Hence, the growth law for $\dot{\bar{a}}$ plays the role of driving $\sigma _{2}(\bar{r})$ $(\forall \bar{r}\in [\bar{a},\bar{b}])$ to approach a specified homeostatic stress $\sigma _{h}$.

A flow chart is presented in Fig. 8(a) of the Appendix to illustrate the update process in the Class 1 of the growth law.

### Class 2: Specifications of ${\delta \tau _{2}}$ and $\dot{\bar{a}}$

Let us consider a growth law in a reduced form of (136) as

143 $$ \delta \tau _{2}(\bar{r}(R)) = \delta \tau _{3}(\bar{r}(R)) = \bar{\mathcal{G}}\left (\boldsymbol{\tau},\boldsymbol{\sigma}\right ) \overset{\mathrm{def}}{=} - \bar{\eta}(\sigma _{2}(r(R))-\bar{\sigma}_{2}), $$

where $\bar{\mathcal{G}}$ is the reduced scalar form of $\boldsymbol{\mathcal{G}}^{*}$ in (136) and $\bar{\eta}$ is a model parameter.

By using a growth law in a form of (143), $\delta \boldsymbol{\tau}$ can be determined from the equilibrium equation (115) and does not explicitly depend on $\bar{\mathbf{L}}$. With $\delta \boldsymbol{\tau }$ obtained, $\mathbf{\bar{L}}$ can be determined by using the constitutive law (130) and the compatibility condition for $\mathbf{\bar{L}}$. Hence, the pair $\delta \boldsymbol{\tau }$ and $\mathbf{\bar{L}}$ may be computed in a semi-coupled way via their connection through the constitutive law (130). The additional growth law as the boundary condition $\dot{\bar{a}}$ (or $\dot{\bar{b}}$) is required for the determination of $\mathbf{\bar{L}}$.

A flow chart is presented in Fig. 8(b) of the Appendix to schematically illustrate the computational process for the Class 2 of the growth law.

#### Compute the Remaining Part of $\delta \boldsymbol{\tau}$ from Equilibrium Equation

For solving the unspecified $\delta \tau _{1}$ from the equilibrium equation (113), we introduce a stress function, $\phi $, so that the increments of residual stress are expressed in terms of $\phi $ as

144 $$ \delta \tau _{1} = \frac{\phi}{\bar{r}^{2}}\quad{\mathrm{and}}\quad \delta \tau _{2} = \frac{1}{2\bar{r}} \frac{\mathrm{d}\phi}{\mathrm{d}\bar{r}}, $$

which automatically satisfies equation (113). The boundary condition (114) becomes

145 $$ \phi (\bar{r}) = 0~~~{\mathrm{at}}~{\bar{r}=\bar{a},\bar{b}}. $$

Substitution of equation (144)_2_ into the left-hand side of equation (143) and integrating the resulting equation yields

146 $$ \phi (\bar{r}) = - 2\bar{\eta}\int _{\bar{a}}^{\bar{r}}(\sigma _{2}(r(R \circ s))-\bar{\sigma}_{2})s\,\mathrm{d}s, $$

on use of the boundary condition $\phi (\bar{a})=0$ in (145), $R\circ s$ being a mapping $\bar{\mathcal{B}}\to \mathcal{B}_{0}$. The other boundary condition $\phi (\bar{b})=0$ simply gives (139). Substituting $\phi $ in (146) into equation (144)_1_ yields $\delta \tau _{1}$. This completes the computation of $\delta \boldsymbol{\tau}$ for updating $\boldsymbol{\tau}$ at instant $t$.

It is clear that (143) may lead to a uniform distribution of $\sigma _{2}(\bar{r})=\bar{\sigma}_{2}$ ($\forall \bar{r}\in [\bar{a}, \bar{b}]$) but it is not necessary that $\bar{\sigma}_{2}=\sigma _{h}$. When $\sigma _{2}(\bar{r})$ achieves an uniform distribution, equation (146) leads to $\delta \boldsymbol{\tau}= \mathbf{0}$. Accordingly, equation (105) indicates that the total increment $\dot{\boldsymbol{\tau}}\,(=\delta \boldsymbol{\tau}+\bar{\mathbf{L}} \boldsymbol{\tau } - (\operatorname{tr}\bar{\mathbf{L}})\boldsymbol{\tau})$ only comprises the convected term $\bar{\mathbf{L}}\boldsymbol{\tau} - (\operatorname{tr}\bar{\mathbf{L}}) \boldsymbol{\tau}$, which is regulated by a separate growth law for $\dot{\bar{a}}$, as shown in the following discussion.

#### Compute $\bar{\mathbf{L}}$ from the Compatibility Condition and Constitutive Law

With $\delta \boldsymbol{\tau}$ obtained, we now compute $\bar{\mathbf{L}}$ using the compatibility condition and the constitutive law. For the problem under consideration, the compatibility condition may be directly derived from equation (44) as

$$ \frac{\mathrm{d}\left (R\bar{\lambda}_{2} \right )}{\mathrm{d}R}= \bar{\lambda}_{1}, $$

which, by taking derivative respect with time, has the rate form

147 $$ \frac{\mathrm{d}\left (R\dot{\bar{\lambda}}_{2} \right )}{\mathrm{d}R}= \dot{\bar{\lambda}}_{1}. $$

By using $\bar{L}_{ii}=\dot{\bar{\lambda}}_{i}/\bar{\lambda}_{i}$ (no summation of $i$), equation (147) may be re-expressed in terms of the components of $\bar{\mathbf{L}}$ as

148 $$ R\frac{\mathrm{d}\bar{L}_{22}}{\mathrm{d}R}= \frac{\bar{\lambda}_{1}}{\bar{\lambda}_{2}}(\bar{L}_{11}-\bar{L}_{22}). $$

On the other hand, the constitutive relation (130) can be rearranged while using the growth law (143) as

149 $$ \bar{L}_{11} = \frac{\gamma _{02}\bar{L}_{22} + \delta \tau _{1}-\bar{\mathcal{G}}}{\gamma _{01}}. $$

Substituting (149) into the right-hand side of equation (148) yields the equation for ${\bar{L}}_{22}$:

150 $$ R\frac{\mathrm{d}\bar{L}_{22}}{\mathrm{d}R}= \frac{\bar{\lambda}_{1}}{\bar{\lambda}_{2}} \frac{(\gamma _{02}-\gamma _{01})\bar{L}_{22} +\delta \tau _{1}-\bar{\mathcal{G}}}{\gamma _{01}}. $$

Integration of (150) with respect to $R$ gives ${\bar{L}}_{22}$, subject to a ‘boundary condition’ for $\bar{L}_{22}$ at, say $R=A$.

For such a ‘boundary condition’, we observe that equations (43) and (44) indicate that

151 $$ \bar{L}_{22}=\frac{\dot{\bar{\lambda}}_{2}}{\bar{\lambda}_{2}} = \left (\frac{\bar{a}}{\bar{r}}\right )^{3} \frac{\dot{\bar{a}}}{\bar{a}} + \frac{\dot{V}(R)}{4\pi \bar{r}^{3}}. $$

Taking the value of the above equation at $R=A$ yields

152 $$ \bar{L}_{22}\big|_{R=A}=\frac{\dot{\bar{a}}}{\bar{a}}. $$

Hence, the boundary condition for $\bar{L}_{22}$ at $R=A$ is determined by the value at the boundary point, $\dot{\bar{a}}$, which is consistent with (141) in Class 1.

It is observed that, in the situation when $\bar{\mathcal{G}}=0$ and $\delta \boldsymbol{\tau}=\mathbf{0}$ when $\sigma _{2}$ approaches a uniform distribution, $\bar{L}_{22}$ does not vanish if $\bar{L}_{22}\big|_{R=A}\neq 0$. Hence, if the uniform $\sigma _{2}$ is different from the specified homeostatic stress $\sigma _{h}$, the change of inner radius $\bar{a}$ may still contribute to a non-zero $\dot{\boldsymbol{\tau}}$ via the convected term, which regulates the evolution of $\sigma _{2}$ toward a specified homeostatic state $\sigma _{h}$. Therefore, the separate growth law of $\bar{L}_{22}\big|_{R=A}$ (or $\dot{\bar{a}}$) for the specification of (152) is a *control condition* rather than a boundary condition. This observation clarifies why the traction-free condition (145) and displacement condition (152) are applied simultaneously on the same shell surface $R=A$.

With $\bar{L}_{22}$ obtained, $\bar{L}_{11}$ in turn can be computed from equation (149), which completes the update of $\boldsymbol{\tau}$ and $\bar{\mathcal{B}}$.

### Class 3: Specifications of $\dot{\tau}_{2}$ and $\dot{\bar{a}}$

In this case $\dot{\tau}_{2}$ is known. Different from the Class 2 growth law, it will need the integration of the fully coupled set of equations including the equilibrium equation (115), the constitutive law (130), and the compatibility condition for $\mathbf{\bar{L}}$ to determine $\dot{\tau}_{1}$ and $\mathbf{\bar{L}}$. A flow chart is presented in Fig. 8(c) of the Appendix to schematically illustrate the update process of the Class 3 growth law.

Here we may specify the reduced form of (109)_1_ as

153 $$ \dot{\tau}_{2}(\bar{r}(R)) = \tilde{\mathcal{G}}\left ( \boldsymbol{\tau},\boldsymbol{\sigma}\right ) \overset{\mathrm{def}}{=} - \tilde{\eta}(\sigma _{2}(r(R))- \tilde{\sigma}_{2}), $$

where $\tilde{\mathcal{G}}$ is the reduced scalar form of $\boldsymbol{\mathcal{G}}$, $\tilde{\eta}$ is a model parameter, and $\tilde{\sigma}_{2}$ is a constant to be determined in the following discussion. By using the specific form of (88), equation (153) may be re-expressed as

154 $$ \delta \tau _{2} = \tilde{\mathcal{G}}\left (\boldsymbol{\tau}, \boldsymbol{\sigma}\right ) + (\bar{L}_{11}+\bar{L}_{22}){\tau _{2} }. $$

On the other hand, by using (153), the constitutive relation (130) can be rearranged in a form similar to (149) as

155 $$ \bar{L}_{11} = \frac{\gamma _{2}\bar{L}_{22}+\delta \tau _{1}-\tilde{\mathcal{G}}}{\gamma _{1}}, $$

where

156 $$ \gamma _{1} = \gamma _{01}+\tau _{2},\quad \gamma _{2} = \gamma _{02}- \tau _{2}. $$

Substituting (155) into the right-hand side of equation (154) and then substituting the resulting expression for $\delta \tau _{2}$ into the equilibrium equation (113) yields

157 $$ \frac{\mathrm{d}\delta \tau _{1}}{\mathrm{d}\bar{r}} + \frac{2}{\bar{r}}\left (\left (1-\frac{\tau _{2}}{\gamma _{1}}\right ) \delta \tau _{1} - \frac{\tau _{2}(\gamma _{1}+\gamma _{2})}{\gamma _{1}}\bar{{L}}_{22} - \left (1-\frac{\tau _{2}}{\gamma _{1}}\right )\tilde{\mathcal{G}} \right ) = 0. $$

By using the relation (52), equation (157) may be transformed into the Lagrangian form

158 $$ \frac{\mathrm{d}\delta \tau _{1}}{\mathrm{d}R} + \mathcal{H}_{\tau}^{*}(R) = 0, $$

where

159 $$ \mathcal{H}_{\tau}^{*}(R) = \frac{\bar{\lambda}_{1}}{\bar{\lambda}_{2}}\frac{2}{R}\left (\left (1- \frac{\tau _{2}}{\gamma _{1}}\right )\delta \tau _{1} - \frac{\tau _{2}(\gamma _{1}+\gamma _{2})}{\gamma _{1}}\bar{{L}}_{22} - \left (1-\frac{\tau _{2}}{\gamma _{1}}\right )\tilde{\mathcal{G}} \right ). $$

Equation (158) provides the first governing equation for solving the pair of unknown variables, $\delta \tau _{1}$ and $\bar{L}_{22}$.

Furthermore, substituting (155) into the right-hand side of the compatibility condition (148) yields the second governing equation for $\delta \tau _{1}$ and $\bar{L}_{22}$:

160 $$ R\frac{\mathrm{d}\bar{L}_{22}}{\mathrm{d}R} = \frac{\bar{\lambda}_{1}}{\bar{\lambda}_{2}} \frac{(\gamma _{2}-\gamma _{1})\bar{{L}}_{22} +\delta \tau _{1}-\tilde{\mathcal{G}}}{\gamma _{1}}. $$

Integration of the set of coupled equations (158) and (160) with respect to $R$ gives $\delta \tau _{1}$ and $\bar{L}_{22}$, which requires determination of $\tilde{\sigma}_{2}$ by using the traction-free condition

161 $$ \mathcal{R}_{\tau}(\tilde{\sigma}_{2})\overset{\mathrm{def}}{=} - \int _{A}^{B}\mathcal{H}_{\tau}^{*}(R)\mathrm{d}{R} = 0, $$

where $\mathcal{R}_{\tau}$ is taken as a function of $\tilde{\sigma}_{2}$.

With $\delta \tau _{1}$ and $\bar{L}_{22}$ thus obtained, $\bar{L}_{11}$ in turn can be computed from equation (155) and the control condition of $\bar{L}_{22}\big|_{R=A}$ (or $\dot{\bar{a}}$). Consequently, $\dot{\bar{p}}$ can be computed using equation (142), which completes the update of $\boldsymbol{\tau}$ and $\bar{\mathcal{B}}$.

### Class 4: Specifications of the Spatial Analytical Form of $\dot{\bar{r}}$

The computational process using the Class 4 growth law is outlined here. Given $\dot{\bar{r}}$, which potentially can be measured by experiments or formulated based on experimental data, $\delta \tau _{1}-\delta \tau _{2}$ can be determined from the constitutive law (130). Then substituting the expression for $\delta \tau _{1}-\delta \tau _{2}$ into the equilibrium equation (113) and integrating this equation determines $\delta \tau _{1}$. Consequently, $\delta \tau _{2}$ can also be determined from the constitutive law (130).

For the purpose of demonstration, here we introduce a method to derive the expression for $\dot{\bar{r}}$ from the change of volume, $\operatorname{tr}\mathbf{\bar{L}}$. We may first specify (112) as a volumetric growth on $\bar{\mathcal{B}}$ as

162 $$ \operatorname{tr}\mathbf{\bar{L}}(\bar{r})= \hat{g}(\bar{r}), $$

where $\hat{g}(\bar{r})$ is a scalar function to be specified. By using $\mathbf{\bar{L}} = \operatorname{diag}[\mathrm{d}\dot{\bar{r}}/\mathrm{d} \bar{r},\dot{\bar{r}}/\bar{r},\dot{\bar{r}}/\bar{r}]$, equation (162) becomes a differential equation for $\dot{\bar{r}}$:

163 $$ \frac{\mathrm{d}\dot{\bar{r}}}{\mathrm{d}\bar{r}}+ \frac{2\dot{\bar{r}}}{\bar{r}}= \hat{g}(\bar{r}). $$

The solution of (163) provides the expression for $\dot{\bar{r}}$, namely

164 $$ \dot{\bar{r}} = \bar{r}^{-2}\left (\beta _{0}+\int ^{\bar{r}}{s}^{2} \hat{g}(s)\mathrm{d}{s}\right ), $$

in which the parameter $\beta _{0}$ is determined by the traction-free boundary condition.

As an example, we take $\hat{g}$ in the form

165 $$ \hat{g}(\bar{r}) = \beta _{1}(\bar{r}^{n}-\bar{b}^{n})+\beta _{2}, $$

which may approximate the volumetric growth by properly fitting the parameter $n$ from data. The coefficients $\beta _{1}$ and $\beta _{2}$ may be determined by homeostatic conditions

166 $$ \beta _{1} = - \eta _{c1}\Big\vert \max _{a\leq r\leq b}(\sigma _{2}(r))- \min _{a\leq r\leq b}(\sigma _{2}(r))\Big\vert \quad{\mathrm{and}} \quad \beta _{2} = - \eta _{c2}(\bar{\sigma}_{2}-\sigma _{h}), $$

where $\eta _{c1}$ and $\eta _{c2}$ are two model parameters. Note the constraints on the model parameters require that either (a) $n>0$ and $\beta _{1}<0$ or (b) $n<0$ and $\beta _{1}>0$ so that the magnitude of volumetric growth decreases monotonically from $\bar{r}=\bar{a}$ to $\bar{r}=\bar{b}$. It is observed that $\beta _{1}$ regulates the process to achieve a uniform distribution of $\sigma _{2}$ (and $\sigma _{3}$) while $\beta _{2}$ controls the process toward a specified homeostatic stress $\sigma _{h}$.

Substituting (165) into (164) yields

167 $$ \dot{\bar{r}} = \beta _{0}\bar{r}^{-2} + \beta _{1}\left ( \frac{\bar{r}^{n+1}}{n+3}-\frac{\bar{b}^{n}\bar{r}}{3}\right ) + \beta _{2}\frac{\bar{r}}{3}, $$

and accordingly

168 $$ \bar{L}_{11} = - 2\beta _{0}\bar{r}^{-3} + \beta _{1}\left ( \frac{(n+1){\bar{r}}^{n}}{n+3}-\frac{\bar{b}^{n}}{3}\right ) + \frac{1}{3}\beta _{2}, $$

169 $$ \bar{L}_{22} = \bar{L}_{33} = \beta _{0}\bar{r}^{-3} + \beta _{1} \left (\frac{\bar{r}^{n}}{n+3}-\frac{\bar{b}^{n}}{3}\right ) + \frac{1}{3}\beta _{2}. $$

Similar to (137) in Class 1, equation (167) or equations (168) and (169) may be considered as a non-local flow rule.

Substitute $\mathbf{\bar{L}}$ in equations (168) and (169) into equation (133). Then we can solve for $\delta \tau _{1}$ from equation (131) subject to the traction-free boundary conditions (114). In such a way, the traction-free condition $\delta \tau _{1}\vert _{\bar{r}=\bar{a}}=0$ at $\bar{r}=\bar{a}$ can be satisfied automatically. The other condition at $\bar{r}=\bar{b}$, i.e. equation (132), is taken as the condition to determine $\beta _{0}$ in equation (164) or (167). With $\delta \tau _{1}$ determined, $\delta \tau _{2}$ can be computed from equation (134).

It should be emphasised that the expression for $\dot{\bar{r}}$ may be obtained from other data or observations different from the change of volume as long as $\dot{\bar{r}}$ can be obtained from the data.

A flow chart is presented in Fig. 9(a) of the Appendix to schematically illustrate the update process in the Class 4 growth law.

### Class 5: Specification of $\operatorname{tr}\mathbf{\bar{L}}$

The rate of volumetric change, $\operatorname{tr}\mathbf{\bar{L}}$, can potentially be measured experimentally. Note that in Class 4 we discuss the non-local growth law (see (164) and (167)) by obtaining $\dot{\bar{r}}$ via the specification of the volumetric change $\operatorname{tr}\mathbf{\bar{L}}$. Here a local form of growth law is proposed, also based on the volumetric change $\operatorname{tr}\mathbf{\bar{L}}$.

For this purpose, the general growth law (112) may be specified as a *scalar* growth law based on the homeostatic condition (135), specifically

170 $$ \operatorname{tr}\mathbf{\bar{L}}(\bar{r}(R))= \bar{g}^{\star}\left ( \boldsymbol{\sigma}(r(R)),\sigma _{h}\right ), $$

where

171 $${\bar{g}}^{⋆}\left(\sigma (r(R)),\sigma_{h}\right)=\{\begin{array}{cc} {\bar{\eta }}^{⋆}(\sigma_{2}(r(R))-\sigma_{h}) & \;\mathrm{if}\;\sigma_{2}(r(R))-\sigma_{h}\geq 0\\ 0 & \;\mathrm{if}\;\sigma_{2}(r(R))-\sigma_{h}<0, \end{array}$$

in which $\bar{\eta}^{\star}$ is a model parameter. By using $\mathbf{\bar{L}} = \operatorname{diag}[\mathrm{d}\dot{\bar{r}}/\mathrm{d} \bar{r},\dot{\bar{r}}/\bar{r},\dot{\bar{r}}/\bar{r}]$, equation (170) becomes a differential equation for $\bar{r}(R)$, i.e.

172 $$ \frac{\mathrm{d}\dot{\bar{r}}}{\mathrm{d}\bar{r}} + \frac{2\dot{\bar{r}}}{\bar{r}} = \bar{g}^{\star}\left ( \boldsymbol{\sigma}(r(R)),\sigma _{h}\right ), $$

which can be solved numerically with the boundary condition for $\dot{\bar{r}}$ at $\bar{r}=\bar{a}$ to be determined.

Consequently, substituting $\mathbf{\bar{L}}=\operatorname{diag}[\mathrm{d}\dot{\bar{r}}/\mathrm{d} \bar{r},\dot{\bar{r}}/\bar{r},\dot{\bar{r}}/\bar{r}]$ into (133) the solution for $\delta \tau _{1}$ is computed from equation (131) subject to the traction-free boundary condition (132) at $\bar{r}=\bar{b}$. The boundary condition (132), in turn, determines $\dot{\bar{a}}$ as the boundary condition of (172). This may be expressed as

173 $$ \mathcal{R}_{a}(\dot{\bar{a}})\overset{\mathrm{def}}{=} - \int _{ \bar{a}}^{\bar{b}}\mathcal{H}_{\tau}(\bar{r})\mathrm{d}{\bar{r}} = 0, $$

noting that $\mathcal{R}_{a}(\dot{\bar{a}})$ is a function of $\dot{\bar{a}}$ via the fact that $\dot{\bar{a}}$ provides the boundary condition to solve equation (172). Hence, a numerical method for solving $\dot{\bar{a}}$ from (173) involves iteratively solving equation (172) corresponding to the iterative value of $\dot{\bar{a}}$.

A flow chart is presented in Fig. 9(b) of the Appendix to illustrate the update process in the Class 5 of the growth law.

### Thermodynamic Constraint Applied to ${\bar{L}}_{22}|_{R=A}$ or $\dot {\bar{a}}$

Note that in Classes 1–3 of the growth laws we need a separate growth law for $\bar{L}_{22}\big|_{R=A}$ (or $\dot{\bar{a}}$) in addition to that of the rate of change (increment) of the residual stress. It is interesting to examine if the residual inequality (24) imposes a meaningful constraint on $\bar{L}_{22}\big|_{R=A}$ when $\delta \boldsymbol{\tau}$ is provided.

#### Thermodynamic Constraints for Spherical Shell Growth

By using (88) and $\mathbf{L}^{\prime}=\mathbf{F}_{e}\bar{\mathbf{L}}\mathbf{F}_{e}^{ \mathrm{T}}$, the inequality (24) becomes

174 $$ \left ( \mathbf{F}_{e}^{\mathrm{T}}\boldsymbol{\sigma}\mathbf{F}_{e} + \left (\frac{\partial \bar{\Psi}}{\partial \boldsymbol{\tau}}: \boldsymbol{\tau}\right ) \bar{\mathbf{I}} - \boldsymbol{\tau} \frac{\partial \bar{\Psi}}{\partial \boldsymbol{\tau}}\right ): \mathbf{\bar{L}} \geq \frac{\partial \bar{\Psi}}{\partial \boldsymbol{\tau}}:\delta \boldsymbol{\tau}. $$

Given $\delta \boldsymbol{\tau}$, the above inequality (174) may be considered as a constraint on $\mathbf{\bar{L}}_{r}$.

According to equations (27) and (34), we have

175 $$ \frac{\partial \bar{\Psi}}{\partial \boldsymbol{\tau}} = \frac{\partial \bar{\Psi}^{\prime}}{\partial \boldsymbol{\tau}} = \frac{1}{2}({\mathbf{C}}_{e}-\bar{\mathbf{I}}). $$

In a spherical shell, the above equation may be expressed in components as

176 $$ \frac{\partial \bar{\Psi}}{\partial \boldsymbol{\tau}} = \frac{\partial \bar{\Psi}^{\prime}}{\partial \boldsymbol{\tau}} = \frac{1}{2}\mathrm{diag}[\lambda ^{-4}-1,\lambda ^{2}-1,\lambda ^{2}-1]. $$

Substituting (176) into the inequality (174) yields

177 $$\begin{aligned} &[\sigma _{1}\lambda ^{-4}+\tau _{2}(\lambda ^{2}-1)]\bar{L}_{11} + [2 \sigma _{2}\lambda ^{2}+(\tau _{1}(\lambda ^{-4}-1)+\tau _{2}( \lambda ^{2}-1))]\bar{L}_{22} \\ &\qquad \qquad \qquad \geq \frac{1}{2}(\lambda ^{-4}-1)\delta \tau _{1} + (\lambda ^{2}-1)\delta \tau _{2}. \end{aligned}$$

#### Thermodynamical Consideration of ${\bar{L}}_{22}|_{R=A}$

The constraint (177) should be satisfied at any point $R\in [A,B]$. Of particular interest is the point $R=A$ where $\dot{\bar{a}}$ as the control condition determines $\dot{\bar{r}}$ in (141) and $\bar{L}_{22}$ in (150) and (160). For this consideration, using equation (130) and the traction-free condition, we may examine the inequality (177) at the point $R=A$ as

178 $$ \mathcal{D}_{A}\overset{\mathrm{def}}{=}\mathbf{n}_{f}\cdot \begin{Bmatrix} \bar{L}_{22} \\ \delta \tau _{2} \end{Bmatrix} \Bigg\vert _{R=A} \geq 0, $$

where $\mathcal{D}_{A}$ is the dissipation increment at the material point $R=A$ and

179 $$ \mathbf{n}_{f} = \begin{Bmatrix} n_{f1} \\ n_{f2} \end{Bmatrix} = \begin{Bmatrix} \sigma _{1}\lambda ^{-4} +2\sigma _{2}\lambda ^{2}+2\tau _{2}( \lambda ^{2}-1) \\ - \dfrac{\sigma _{1}\lambda ^{-4}+\tau _{2}(\lambda ^{2}-1)}{\gamma _{01}} - (\lambda ^{2}-1) \end{Bmatrix} \Bigg\vert _{R=A}. $$

It is interesting that the constraint (178) is in a form analogous to the flow rule in plasticity theory where $\mathbf{n}_{f}$ represents the flow direction. The inequality (178) is considered as a constraint on $\bar{L}_{22}\big\vert _{R=A}$ given $\delta \tau _{2}\big\vert _{R=A}$. We shall discuss how to directly propose a growth law for $\bar{L}_{22}\vert _{R=A}$ in Classes 1–3 to strictly satisfy the constraint (178) *a priori*.

A ‘non-associated flow rule’ is proposed as

180 $$\begin{array}{c} \max_{{\bar{L}}_{22}{|}_{R=A}}D_{A}\\ such\;that\;D_{A}\geq 0\;\mathrm{and}\;|{\bar{L}}_{22}{|}_{R=A}|\leq {\dot{\lambda }}_{f}, \end{array}\}$$

where $\dot{\lambda}_{f}\,(\geq 0)$ is the analogue of a plastic multiplier and plays the role of controlling the magnitude of $\bar{L}_{22}\vert _{R=A}$. The solution of the optimum problem (180) is

181 $$\begin{array}{c} {\bar{L}}_{22}{|}_{R=A}=\mathrm{sign}(n_{f1}){\dot{\lambda }}_{f}\\ such\;that\;{\dot{\lambda }}_{f}\geq |\frac{n_{f2}}{n_{f1}}\delta \tau_{2}{|}_{R=A}|\;\mathrm{if}\;\delta \tau_{2}\;n_{f2}<0. \end{array}\}$$

Based on (181), the growth law for $\bar{L}_{22}\big|_{R=A}$ is proposed as

182 $${\bar{L}}_{22}{|}_{R=A}=\{\begin{array}{c} \mathrm{sign}(n_{f1}){\dot{\lambda }}_{f}\;\mathrm{if}\;{\bar{D}}_{A}({\dot{\lambda }}_{f})\geq 0\\ -c_{\lambda }\frac{n_{f2}}{n_{f1}}\delta \tau_{2}{|}_{R=A}\;\mathrm{if}\;{\bar{D}}_{A}({\dot{\lambda }}_{f})<0, \end{array}$$

where

183 $$ \dot{\lambda}_{f}=\Bigg\vert \frac{c_{r}}{\bar{a}}\left (1- \frac{\bar{\sigma}_{2}}{\sigma _{h}}\right )\Bigg\vert ,\quad \bar{\mathcal{D}}_{A}(\dot{\lambda}_{f}) = \mathbf{n}_{f}\cdot \begin{Bmatrix} \mathrm{sign}(n_{f1})\dot{\lambda}_{f} \\ \delta \tau _{2}\big\vert _{R=A} \end{Bmatrix} , $$

and $c_{\lambda}\,(>1)$ is a control parameter. The growth increment $\begin{Bmatrix} \bar{L}_{22} , & \delta \tau _{2} \end{Bmatrix} ^{\mathrm{T}} \big\vert _{R=A}$ computed from (182) can strictly satisfy the thermodynamic constraint *a priori*.

## Numerical Treatment of the Growth Models for an Incompressible Elastic Spherical Shell

For the computation purposes, the whole reference domain $R\in [A,B]$ is discretised into $n_{e}$ elements. Here the length of each element is $\Delta{R}=R/n_{e}$ if the reference domain is divided uniformly. By using the Lagrangian description, each node, $I\in [1,n_{e}+1]$, on this mesh represents a *material* point. The current states of all variables, $r$, $\bar{r}$, $\mathbf{F}$, $\bar{\mathbf{F}}$, $\mathbf{F}_{e}$, $\boldsymbol{\sigma}$, and $\boldsymbol{\tau}$, are computed and stored at each and every node.

The $i_{e}$th element occupies a domain $R\in [R_{i_{e}},R_{i_{e}+1}]\subset \mathcal{B}_{0}$ where $i_{e}$ and $i_{e}+1$ are two endpoints of the element and $R_{i_{e}}$ and $R_{i_{e}+1}$ stand for the values of $R$ at the nodal points $I=i_{e}$ and $I=i_{e}+1$, respectively. The computation of growth is conducted on the unloaded configuration $\bar{\mathcal{B}}$ where the nodes of the ${i_{e}}$th element are mapped to the spatial locations $\bar{r}_{i_{e}+1}=\bar{r}(R_{i_{e}+1})$ and $\bar{r}_{i_{e}}=\bar{r}(R_{i_{e}})$, respectively. The Cauchy stress is computed on the current configuration ℬ where the nodes of the ${i_{e}}$th element are located at the spatial points $r_{i_{e}+1}= r(R_{i_{e}+1})$ and $r_{i_{e}}= r(R_{i_{e}})$, respectively.

We consider such a process: at the initial time $t=t_{0}$, the shell is free from residual stress and is purely elastically deformed due to external loads, then the homeostatic condition (135) causes the growth to take place continuously until the condition is satisfied. To model this process, we shall start from computing the initial elastically-deformed configuration and the stresses within it. This provides the initial input to compute the initiation of growth. Then, at the beginning of each time step, say $t$, the state variables, $\{\boldsymbol{\tau}, \bar{\mathcal{B}}\}$ and $\{\boldsymbol{\sigma},{\mathcal{B}}\}$, are obtained from the computation of the last time step. By using the stress-driven growth law depending on $\boldsymbol{\sigma}$ and $\boldsymbol{\tau}$, the increment, $\delta \boldsymbol{\tau}$ and $\bar{\mathbf{L}}$, may be computed, while the condition (178) provides a practical method either to satisfy the thermodynamical constraint *a priori* or to check it numerically *a posteriori*. Consequently, an explicit forward update algorithm is used to update $\boldsymbol{\tau}$ and $\bar{\mathcal{B}}$ at the end of the time step $t+\Delta t$. We then compute the elastic response $\mathbf{F}_{e}$ and Cauchy stress $\boldsymbol{\sigma}$ based on the hyperelastic formulation (23) or (66) on the configuration $\bar{\mathcal{B}}$. This completes the update for the time step $t\to t+\Delta t$. The update process continues until a homeostatic state is met.

### Algorithms for Computing Increments of the Growth Variables

Given Cauchy stress $\boldsymbol{\sigma}$ and two growth variables, $\boldsymbol{\tau}$ and $\bar{\mathcal{B}}$, at time $t\,(>t_{0})$, we discuss the process for computing the increments of the two growth variables for the time $t+\Delta t$.

#### Class 1 Growth Update Algorithm

According to (137), at all nodal points $I\in [1,n_{e}+1]$, we have the increments of $\boldsymbol{\tau}$:

184 $$ \delta \tau _{1}(\bar{r}_{I})=\delta \nu (\bar{r}_{I}-\bar{a})( \bar{r}_{I}-\bar{b}),\quad \delta \tau _{2}(\bar{r}_{I}) = \delta \nu [4\bar{r}_{I}^{2}-3(\bar{a}+\bar{b})\bar{r}_{I}+2\bar{a}\bar{b}]/2. $$

The average value of $\sigma _{2}$ in (138) is computed as

185 $$ \bar{\sigma}_{2}=\frac{1}{b^{2}-a^{2}}\sum _{I= 1}^{ n_{e}}(\sigma _{2}(r_{i_{e}})+ \sigma _{2}(r_{i_{e}+1}))r_{i_{e}}^{\mathrm{mid}}(r_{i_{e}+1}-r_{i_{e}}), $$

where $r_{i_{e}}^{\mathrm{mid}}=\frac{1}{2}( r_{i_{e}}+ r_{i_{e}+1})$. This gives $\delta \nu $ in equation (138).

The ordinary differential equation (141) is approximated by the discrete format:

186 $$ \gamma _{01}(\bar{r}_{i_{e}}^{\mathrm{mid}}) \frac{\dot{\bar{r}}_{i_{e}+1}-\dot{\bar{r}}_{i_{e}}}{\bar{r}_{i_{e}+1}-\bar{r}_{i_{e}}} - \gamma _{02}(\bar{r}_{i_{e}}^{\mathrm{mid}}) \frac{\dot{\bar{r}}_{i_{e}+1}+\dot{\bar{r}}_{i_{e}}}{2\bar{r}_{i_{e}}^{\mathrm{mid}}} + \delta \nu \frac{\bar{r}_{i_{e}}^{\mathrm{mid}}[2\bar{r}_{i_{e}}^{\mathrm{mid}}-(\bar{a}+\bar{b})]}{2} = 0, $$

where the midpoint rule $\bar{r}_{i_{e}}^{\mathrm{mid}}=\frac{1}{2}(\bar{r}_{i_{e}}+\bar{r}_{i_{e}+1})$ is used. From (186) we have the spatial forward-format to compute $\dot{\bar{r}}$ from $i_{e}=1$:

187 $$ \begin{aligned}[b] \dot{\bar{r}}_{i_{e}+1} &= \left ( \frac{\gamma _{01}(\bar{r}_{i_{e}}^{\mathrm{mid}})}{\bar{r}_{i_{e}+1}-\bar{r}_{i_{e}}} - \frac{\gamma _{02}(\bar{r}_{i_{e}}^{\mathrm{mid}})}{2\bar{r}_{i_{e}}^{\mathrm{mid}}} \right )^{-1} \left (\left ( \frac{\gamma _{01}(\bar{r}_{i_{e}}^{\mathrm{mid}})}{\bar{r}_{i_{e}+1}-\bar{r}_{i_{e}}} + \frac{\gamma _{02}(\bar{r}_{i_{e}}^{\mathrm{mid}})}{2\bar{r}_{i_{e}}^{\mathrm{mid}}} \right )\dot{\bar{r}}_{i_{e}}\right. \\ &\quad{} - \left.\delta \nu \frac{\bar{r}_{i_{e}}^{\mathrm{mid}}[2\bar{r}_{i_{e}}^{\mathrm{mid}}-(\bar{a}+\bar{b})]}{2} \right ). \end{aligned} $$

Equation (187) determines $\dot{\bar{r}}$ as a function of $\bar{r}$ given the boundary condition for $\dot{\bar{a}}$. Equations (184) and (187) complete the computation of the increments of $\boldsymbol{\tau}$ and $\bar{\mathcal {B}}$.

#### Class 2 Growth Update Algorithm

The stress function $\phi $ may be computed by using the discrete integration format of (146),

188 $$ \phi (\bar{r}_{i_{e}+1})=\phi (\bar{r}_{i_{e}}) - 2\bar{\eta}\left \{ \left \{(\sigma _{2}-{\bar{\sigma}}_{2})\bar{r}\right \} \bigg\vert _{ \bar{r}=\bar{r}_{i_{e}}^{\mathrm{mid}}}({\bar{r}_{i_{e}+1}-\bar{r}_{i_{e}}}) \right \}, $$

for all elements $i_{e}\in [1,n_{e}]$ starting from $i_{e}=1$ where the boundary condition (145) indicates $\phi (\bar{r}_{i_{e}})\vert _{i_{e}=1}=\phi (\bar{a})=0$. The average stress $\bar{\sigma}_{2}$ is computed using (185). With $\phi (\bar{r}_{i_{e}+1})$ obtained, $\delta \tau _{1}(\bar{r}_{i_{e}+1})$ and $\delta \tau _{2}(\bar{r}_{i_{e}+1})$ can be computed from (144)_1_ and (143), respectively, for all elements. This completes the computation of $\delta \boldsymbol{\tau}$ at all nodal points $I\in [1,n_{e}+1]$.

Consequently, ${\bar{L}}_{22}$ may be computed from equation (150). This can be done by using the central difference approximation of (150) in, say the $i_{e}$th element, in a form such as

189 $$ \frac{{\bar{L}}_{22}(\bar{r}_{i_{e}+1})-{\bar{L}}_{22}(\bar{r}_{i_{e}})}{\Delta R} = c_{1} \frac{{\bar{L}}_{22}(\bar{r}_{i_{e}+1})+{\bar{L}}_{22}(\bar{r}_{i_{e}})}{2} + c_{2}, $$

where

$$ c_{1} = \left \{\frac{\bar{\lambda}_{1}}{R\bar{\lambda}_{2}} \frac{\gamma _{02}-\gamma _{01}}{\gamma _{01}} \right \}\Bigg\vert _{R_{i_{e}}^{ \mathrm{mid}}},\quad c_{2} = \left \{ \frac{\bar{\lambda}_{1}}{R\bar{\lambda}_{2}} \frac{\delta \tau _{1}-\delta \tau _{2}}{\gamma _{01}}\right \} \Bigg\vert _{R_{i_{e}}^{\mathrm{mid}}}, $$

in which $R_{i_{e}}^{\mathrm{mid}}=\frac{1}{2}(R_{i_{e}}+R_{i_{e}+1})$. From equation (189), the spatial forward format for computing ${\bar{L}}_{22}$ is

190 $$ \bar{L}_{22}(\bar{r}_{i_{e}+1}) = \left (\frac{1}{\Delta R}- \frac{c_{1}}{2}\right )^{-1} \left (\left (\frac{1}{\Delta R} + \frac{c_{1}}{2}\right )\bar{L}_{22}(\bar{r}_{i_{e}})+c_{2}\right ). $$

This completes the update of $\boldsymbol{\tau}$ and $\bar{\mathcal{B}}$.

#### Class 3 Growth Update Algorithm

The governing equations (158) and (160) may be expressed together in matrix form as

191 $$ \mathbf{M}\frac{\mathrm{d}\mathbf{y}}{\mathrm{d}R}+\mathbf{A} \mathbf{y} = \mathbf{f}, $$

where

$$ \mathbf{M}=R\mathbf{1},\quad \mathbf{A}= \begin{bmatrix} A_{11} & A_{12} \\ A_{21} & A_{22} \end{bmatrix} ,\quad \mathbf{y} = \begin{Bmatrix} \delta \tau _{1} \\ \bar{L}_{22} \end{Bmatrix} ,\quad \mathbf{f}= \begin{Bmatrix} f_{1} \\ f_{2} \end{Bmatrix} , $$

in which $[\mathbf {1}]_{ij}=\delta _{ij}$,

$$ A_{11} = 2\frac{\bar{\lambda}_{1}}{\bar{\lambda}_{2}}\left (1- \frac{\tau _{2}}{\gamma _{1}}\right ),\quad A_{12} = - 2 \frac{\bar{\lambda}_{1}}{\bar{\lambda}_{2}} \frac{\tau _{2}(\gamma _{1}+\gamma _{2})}{\gamma _{1}}, $$

$$ A_{21} = - \frac{\bar{\lambda}_{1}}{\bar{\lambda}_{2}\gamma _{1}}, \quad A_{22} = \frac{\bar{\lambda}_{1}(\gamma _{1}-\gamma _{2})}{\bar{\lambda}_{2}\gamma _{1}} = A_{21}(\gamma _{2}-\gamma _{1}), $$

$$ f_{1} = 2\frac{\bar{\lambda}_{1}}{\bar{\lambda}_{2}}\left (1- \frac{\tau _{2}}{\gamma _{1}}\right )\tilde{\mathcal{G}} = A_{11} \tilde{\mathcal{G}},\quad f_{2} = - \frac{\bar{\lambda}_{1}}{\bar{\lambda}_{2}\gamma _{1}} \tilde{\mathcal{G}} = A_{21}\tilde{\mathcal{G}}. $$

By using the midpoint rule of approximation, equation (191) may be expressed in the discrete format,

192 $$ \mathbf{M}_{i_{e}+\frac{1}{2}} \frac{\mathbf{y}_{i_{e}+1}-\mathbf{y}_{i_{e}}}{\Delta R} + \mathbf{A}_{i_{e}+ \frac{1}{2}}\frac{\mathbf{y}_{i_{e}+1}+\mathbf{y}_{i_{e}}}{2} = \mathbf{f}_{i_{e}+\frac{1}{2}}, $$

where $i_{e}+\frac{1}{2}$ stands for the midpoint $R_{i_{e}}^{\mathrm{mid}}$ between the nodal points, $i_{e}$ and $i_{e}+1$. Solving (192) yields the spatial forward format for computing $\mathbf{y}$:

193 $$ \mathbf{y}_{i_{e}+1} = \left [\mathbf{1} + \frac{\Delta R}{2R_{i_{e}+\frac{1}{2}}}\mathbf{A}_{i_{e}+\frac{1}{2}} \right ]^{-1} \left \{\left [\mathbf{1} - \frac{\Delta R}{2R_{i_{e}+\frac{1}{2}}}\mathbf{A}_{i_{e}+\frac{1}{2}} \right ]\mathbf{y}_{i_{e}} +\frac{\Delta R}{R_{i_{e}+\frac{1}{2}}} \mathbf{f}_{i_{e}+\frac{1}{2}} \right \}. $$

At the starting point $R=A$ we have

$$ \mathbf{y}(A)= \begin{Bmatrix} \delta \tau _{1}(A) \\ \bar{L}_{22}(A) \end{Bmatrix} = \begin{Bmatrix} 0 \\ \dot{\bar{a}}/\bar{a} \end{Bmatrix} , $$

where $\dot{\bar{a}}$ is discussed in Sect. 8.6.2. The exact value of $\tilde{\sigma}_{2}$ in $\tilde{\mathcal{G}}$ (see equation (153)) is determined by the traction-free condition (161). Hence, the computation of $\mathbf{y}$ using (193) involves the iterative computation of $\tilde{\sigma}_{2}$ to meet the condition (161).

#### Class 4 Growth Update Algorithm

According to equations (167)–(169), the global increment of $\bar{\mathcal{B}}$ is computed at all nodal points $I\in [1,n_{e}+1]$:

194 $$ \dot{\bar{r}}_{I} = \beta _{0}{\bar{r}_{I}}^{-2} + \beta _{1}\left ( \frac{{\bar{r}_{I}}^{n+1}}{n+3}-\frac{\bar{b}^{n}\bar{r}_{I}}{3} \right ) +\beta _{2}\frac{\bar{r}_{I}}{3}, $$

and

195 $$ \bar{L}_{11} = - 2\beta _{0}{\bar{r}_{I}}^{-3} +\beta _{1}\left ( \frac{(n+1){\bar{r}_{I}}^{n}}{n+3}-\frac{\bar{b}^{n}}{3}\right ) + \frac{1}{3}\beta _{2}, $$

196 $$ \bar{L}_{22} = \bar{L}_{33} = \beta _{0}{\bar{r}_{I}}^{-3} + \beta _{1} \left (\frac{{\bar{r}}_{I}^{n}}{n+3}-\frac{\bar{b}^{n}}{3}\right ) + \frac{1}{3}\beta _{2}. $$

The coefficients $\beta _{1}$ and $\beta _{2}$ are computed by (166). The other parameter $n$ can be fitted from experimental data while $\beta _{0}$ is determined by the traction-free boundary condition as discussed below.

$\delta \tau _{1}$ is computed from equation (131) in the discrete form:

197 $$ \delta \tau _{1}(\bar{r}_{i_{e}+1})=\delta \tau _{1}(\bar{r}_{i_{e}}) - \mathcal{H}_{\tau}(\bar{r}_{i_{e}}^{\mathrm{mid}}) (\bar{r}_{i_{e}+1}- \bar{r}_{i_{e}}), $$

while the traction-free condition (132) is approximated as

198 $$ \sum _{i_{e}=1}^{n_{e}}\mathcal{H}_{\tau}(\bar{r}_{i_{e}}^{ \mathrm{mid}})(\bar{r}_{i_{e}+1}-\bar{r}_{i_{e}}) = 0. $$

By substituting equations (195) and (196) into equation (198), the coefficient $\beta _{0}$ in the expression of $\dot{\bar{r}}$ is computed as

$$ \beta _{0} = \frac{\sum _{i_{e}=1}^{n_{e}}\left (\gamma _{01}(\bar{r}_{i_{e}}^{\mathrm{mid}})\left (\beta _{1} \left (\frac{(n+1)(\bar{r}_{i_{e}}^{\mathrm{mid}})^{n}}{n+3}-\frac{\bar{b}^{n}}{3}\right )+\frac{\beta _{2}}{3}\right ) - \gamma _{02}(\bar{r}_{i_{e}}^{\mathrm{mid}})\left (\beta _{1}\left (\frac{(\bar{r}_{i_{e}}^{\mathrm{mid}})^{n}}{n+3}-\frac{\bar{b}^{n}}{3}\right )+\frac{\beta _{2}}{3}\right )\right )\frac{\bar{r}_{i_{e}+1}-\bar{r}_{i_{e}}}{\bar{r}_{i_{e}}^{\mathrm{mid}}}}{\sum _{i_{e}=1}^{n_{e}}\frac{2\gamma _{01}(\bar{r}_{i_{e}}^{\mathrm{mid}})+\gamma _{02}(\bar{r}_{i_{e}}^{\mathrm{mid}})}{(\bar{r}_{i_{e}}^{\mathrm{mid}})^{4}}(\bar{r}_{i_{e}+1}-\bar{r}_{i_{e}})}. $$

With $\beta _{0}$ obtained, $\delta \tau _{1}$ at all nodes can be computed from (197). Consequently, $\delta \tau _{2}$ is computed from the constitutive relation (130) as

199 $$ \delta \tau _{2}(\bar{r}_{i_{e}+1}) = \delta \tau _{1}(\bar{r}_{i_{e}+1})- \frac{\bar{r}_{i_{e}+1}}{2}\mathcal{H}_{\tau}(\bar{r}_{i_{e}+1}). $$

This completes the update of $\boldsymbol{\tau}$ and $\bar{\mathcal{B}}$.

#### Class 5 Growth Update Algorithm

By using mid-point rule in an element $i_{e}\in [1,n_{e}]$, equation (172) may be approximated in discrete form as

200 $$ \frac{\dot{\bar{r}}_{i_{e}+1} - \dot{\bar{r}}_{i_{e}}}{\bar{r}_{i_{e}+1}-\bar{r}_{i_{e}}} + 2 \frac{\dot{\bar{r}}_{i_{e}+1}+\dot{\bar{r}}_{i_{e}}}{\bar{r}_{i_{e}+1}+\bar{r}_{i_{e}}} = \frac{\bar{g}^{\star}\left (\boldsymbol{\sigma}(r_{i_{e}+1}),\sigma _{h}\right )+\bar{g}^{\star}\left (\boldsymbol{\sigma}(r_{i_{e}}),\sigma _{h}\right )}{2}. $$

After some algebraic manipulation, equation (200) gives the forward format for computing $\dot{\bar{r}}_{i_{e}+1}$ as

201 $$ \dot{\bar{r}}_{i_{e}+1} = c_{3}\dot{\bar{r}}_{i_{e}}+c_{4}(\sigma _{h}), $$

where the coefficients, $c_{3}$ and $c_{4}$, are

$$ c_{3}= \frac{-\bar{r}_{i_{e}+1}+3\bar{r}_{i_{e}}}{3\bar{r}_{i_{e}+1}-\bar{r}_{i_{e}}} $$

and

$$ c_{4}(\sigma _{h})= \frac{\bar{(}g)^{\star}\left (\boldsymbol{\sigma}(r_{i_{e}+1}),\sigma _{h}\right )+\bar{g}^{\star}\left (\boldsymbol{\sigma}(r_{i_{e}}),\sigma _{h}\right )}{2} \frac{\bar{r}_{i_{e}+1}+\bar{r}_{i_{e}}}{3\bar{r}_{i_{e}+1}-\bar{r}_{i_{e}}}( \bar{r}_{i_{e}+1}-\bar{r}_{i_{e}}), $$

in which $c_{4}(\sigma _{h})$ is a function of $\sigma _{h}$.

With $\dot{\bar{r}}_{i_{e}+1}$ obtained, $\mathbf{\bar{L}}(\bar{r}_{i_{e}+1})$ is computed from the discrete form of $\mathbf{\bar{L}}=\operatorname{diag}[\mathrm{d}\dot{\bar{r}}/\mathrm{d} \bar{r},\dot{\bar{r}}/\bar{r}, \dot{\bar{r}}/\bar{r}]$ as

202 $$ \bar{L}_{11}(\bar{r}_{i_{e}+1})\approx \frac{\dot{\bar{r}}_{i_{e}+1}-\dot{\bar{r}}_{i_{e}}}{{\bar{r}}_{i_{e}+1}-{\bar{r}}_{i_{e}}} \equiv c_{5}\dot{\bar{r}}_{i_{e}}+c_{6}(\sigma _{h}), $$

203 $$ \bar{L}_{22}(\bar{r}_{i_{e}+1}) = \bar{L}_{33}(\bar{r}_{i_{e}+1}) \approx \frac{\dot{\bar{r}}_{i_{e}+1}+\dot{\bar{r}}_{i_{e}}}{\bar{r}_{i_{e}+1}+\bar{r}_{i_{e}}} \equiv c_{7}\dot{\bar{r}}_{i_{e}}+c_{8}(\sigma _{h}), $$

where

$$ c_{5} =\frac{c_{3}-1}{\bar{r}_{i_{e}+1}-\bar{r}_{i_{e}}},\quad c_{6}( \sigma _{h}) = \frac{c_{4}(\sigma _{h})}{\bar{r}_{i_{e}+1}-\bar{r}_{i_{e}}}, $$

$$ c_{7} =\frac{c_{3}+1}{\bar{r}_{i_{e}+1}+\bar{r}_{i_{e}}},\quad c_{8}( \sigma _{h}) = \frac{c_{4}(\sigma _{h})}{\bar{r}_{i_{e}+1}+\bar{r}_{i_{e}}}. $$

It is observed from equations (201)–(203) that $\dot{\bar{r}}_{i_{e}+1}$ and $\mathbf{\bar{L}}(\bar{r}_{i_{e}+1})$ are all linear functions of $\dot{\bar{r}}_{i_{e}}$, which provides a convenient spatial forward format for the computation.

With $\mathbf{\bar{L}}$ obtained, $\delta \tau _{1}$ may be computed using the same format as (197) with $\bar{L}_{11}$ and $\bar{L}_{22}$ computed from (202) and (203). Then $\delta \tau _{2}(\bar{r}_{i_{e}+1})$ is computed using the same format as (199). Noting that at the starting point $R=A$, $\dot{\bar{r}}_{i_{e}}\vert _{i_{e}=1} = \dot{\bar{a}}$ is unknown and needs to be computed by using the condition (173).

If $\bar{r}$ is frozen at the time $t$ (as an approximation of $\bar{\mathcal{B}}$ at time $t+\Delta t$) when computing the increments for $t+\Delta t$, equations (201)–(203) provide a set of explicit formulations of the increments to (173) for computing $\dot{\bar{a}}$ as the root of the equation:

204 $$ \mathcal{R}_{a}^{t}(\dot{\bar{a}})=-\int _{\bar{a}(t)}^{\bar{b}(t)} \mathcal{H}_{\tau} \mathrm{d}{\bar{r}}(t) {\approx} \sum _{i_{e}=1}^{n_{e}} \left \{\mathcal{H}_{\tau}\Big\vert _{\bar{r}=\bar{r}_{i_{e}}^{ \mathrm{mid}}}(\bar{r}_{i_{e}+1}-\bar{r}_{i_{e}}) \right \} \bigg\vert _{\bar{r}={\bar{r}}(t)}=0. $$

Equation (204) can be solved by standard algorithms such as the bisection method. This completes the computation of $\delta \boldsymbol{\tau}$ and $\mathbf{\bar{L}}$ for $t+\Delta t$.

### Updating $\boldsymbol{\tau}$ and $\bar{\mathcal{B}}$

With $\delta \boldsymbol{\tau}$ and $\mathbf{\bar{L}}$ obtained from any of the Class1–5 models, it is straightforward to update $\boldsymbol{\tau}$ by using equation (88) as

205 $$ \boldsymbol{\tau}\big\vert _{t+\Delta t}=\boldsymbol{\tau}\big\vert _{t}+( \delta \boldsymbol{\tau} - \operatorname{tr}(\mathbf{\bar{L}}) \boldsymbol{\tau} + \mathbf{\bar{L}}\boldsymbol{\tau})\big\vert _{t} \Delta t. $$

The general format for updating $\bar{\mathcal{B}}$ is written

$$ \bar{\mathbf{F}}\big\vert _{t+\Delta t}=\left (\bar{\mathbf{I}} + \Delta t\mathbf{\bar{L}}\big\vert _{t}\right )\bar{\mathbf{F}} \big\vert _{t}. $$

Note that $\bar{\mathbf{F}}$ is a compatible deformation gradient. Hence, in the case of spherical shell, updating of $\bar{\mathcal{B}}$ just involves updating $\bar{r}$. Using (45)_2_, the increment of $\bar{r}(R)$ is computed as

206 $$ \dot{\bar{r}}=\bar{r}^{-2}\left (\bar{a}^{2}\dot{\bar{a}} + \frac{\dot{V}(R,t)}{4\pi}\right ), $$

in which

207 $$ \dot{V}(R,t) = 4\pi \int _{A}^{R} S^{2}\operatorname{tr}\mathbf{\bar{L}}(S,t) \bar{J}(S,t)\mathrm{d}S. $$

$\dot{V}(R,t)$ at all nodal points $I\in [1,n_{e}+1]$ can be computed by using a spatial forward format of equation (207):

208 $$ \dot{V}(R_{i_{e}+1},t)=\dot{V}(R_{i_{e}},t)+4\pi \left \{ R^{2} \operatorname{tr}\mathbf{\bar{L}}(R,t) \bar{J}(R,t)\right \}\Big\vert _{R=R_{i_{e}}^{ \mathrm{mid}}}\Delta R. $$

Then $\bar{r}$ can be updated for time $t+\Delta t$ using the discrete format of (206),

209 $$ \bar{r}\big\vert _{t+\Delta t} = \bar{r}\big\vert _{t}+\dot{\bar{r}} \big\vert _{t}\Delta t = \bar{r}\big\vert _{t}+\bar{r}^{-2}\left (( \bar{a}^{2}\dot{\bar{a}})\big\vert _{t} +\frac{\dot{V}(R,t)}{4\pi} \right )\Delta t. $$

### Computing Cauchy Stress on the Configuration ℬ During Growth

With $\boldsymbol{\tau}$ and $\bar{\mathcal{B}}$ updated, $\boldsymbol{\sigma}$ and ℬ at time $t+\Delta t$ can be computed as a purely elastic problem. This elastic problem can be completely solved if $a(t+\Delta t)$ is determined by (62).

The discrete format of equation (62) is written as

210 $$ \mathcal{R}_{L}^{h}(a) = \sum _{i_{e}=1}^{n_{e}}\left \{\mathcal{H}( \lambda )\frac{\varkappa \Delta R}{\lambda ^{3}R}\right \}\bigg\vert _{R=R_{i_{e}}^{ \mathrm{mid}}}+P=0, $$

where

$$ \mathcal{H}(\lambda )=\lambda \frac{\partial \hat{\Psi}(\lambda ,\tau _{1},\tau _{2})}{\partial \lambda}, \quad \varkappa =\frac{\bar{J}}{\bar{\lambda}_{2}^{3}}. $$

Equation (210) is the nonlinear equation of the current internal radius $a$ which can be solved by a numerical method.

With $a$ obtained, $r$ may be updated by using equation (45) as

211 $$ r=\left (a^{3} +\frac{3V(R,t+\Delta t)}{4\pi}\right )^{1/3}, $$

where $V(R,t+\Delta t)$ is computed by using the increment in equation (208). Then the discrete forms of equations (60) and (55) are used to compute the Cauchy stress as follows:

212 $$ \sigma _{1}(r_{i_{e}+1}) \approx \sigma _{1}(r_{i_{e}}) + \left \{ \frac{\mathcal{H}(\lambda )}{r}\right \}\Bigg\vert _{r=r_{i_{e}}^{ \mathrm{mid}}}({r_{i_{e}+1}-r_{i_{e}}}), $$

213 $$ \sigma _{2}(r_{i_{e}+1}) = \sigma _{1}(r_{i_{e}+1}) + \frac{1}{2} \mathcal{H}(\lambda (r_{i_{e}+1})). $$

## Numerical Case Studies of Growth in an Incompressible Elastic Spherical Shell

Numerical studies for the growth laws of Classes 1–5 are now presented. We consider a spherical shell with the reference inner and outer radii, $A=1.0\text{ mm}$ and $B=1.1\text{ mm}$, respectively. The reference domain is uniformly divided into $n_{e}=100$ elements. The traction boundary condition is of typical blood pressure, $\sigma _{a}=0.01\text{ MPa}$ and $\sigma _{b}=0$, respectively. The elastic material parameter is set as $\mu =0.1\text{ MPa}$, which is in the typical range for an artery wall tissue. The homeostatic stress is assumed as $\sigma _{h}=0.085\text{ MPa}$ which is slightly higher than the initial averaged value of the circumferential stress $\sigma _{2}$ to represent, say, the effect of hypertension.

The processes of growth to achieve the homeostatic states mentioned in the beginning of Sect. 9 are computed for an *assumed* duration of $t\in [0,1.0]\text{ Ms}$ where 1.0 Ms $=10^{6}$ seconds $\approx 12$ days. Hence this is a slow growth process consistent with the assumption that inertial forces are ignorable.

### Class 1 Growth Law

The values of the model parameters in equations (138) and (183) are set as $\eta _{\nu}=5.0\times 10^{-2}\text{ mm}^{-2}$ and $c_{r}=2.0\times 10^{-4}\text{ mm}$, respectively.

**Fig. 2:**
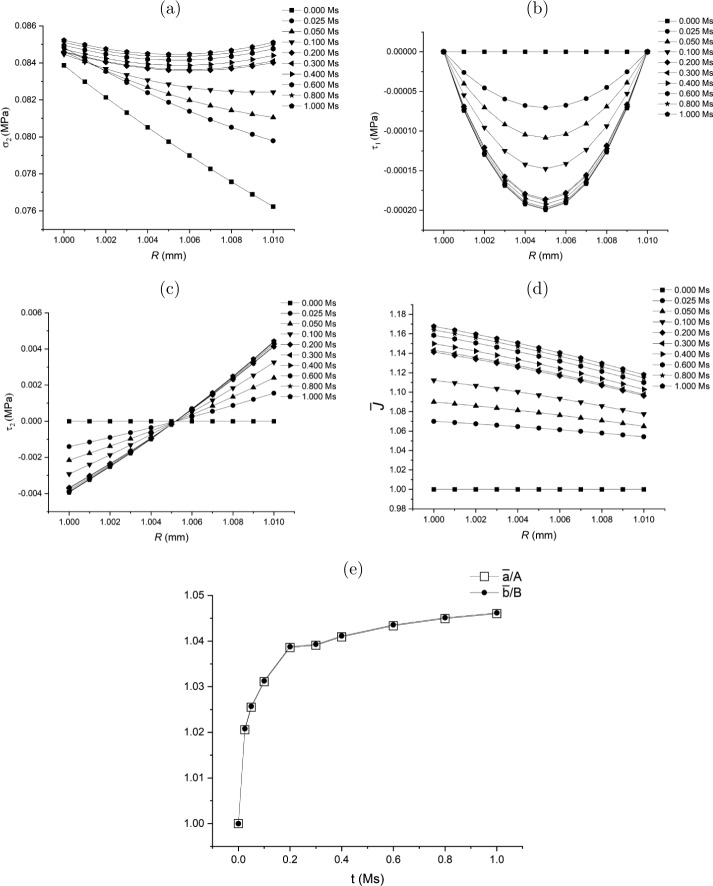
*Class 1*: The temporal development ($t\in [0,1.0]\text{ Ms}$) of (a) the circumferential stress $\sigma _{2}$, (b) the radial residual stress $\tau _{1}$, (c) the circumferential residual stress $\tau _{2}$, (d) the volumetric growth $\bar{J}$, (e) the inner and outer radii of the unloaded configuration, $\bar{a}$ and $\bar{b}$, normalised by their initial values, $A$ and $B$, respectively

The temporal development ($t\in [0,1.0]$ Ms) of the circumferential stress $\sigma _{2}$ is shown in Fig. 2(a). The figure shows that the distribution of the circumferential stress $\sigma _{2}$ approaches the specified homeostatic state, $\sigma _{h}=0.085\text{ MPa}$ if measured by a 2-norm, $\vert \vert \sigma _{2}\vert \vert _{2}$, defined as

214 $$ \vert \vert \sigma _{2}\vert \vert _{2}\overset{\mathrm{def}}{=} \frac{2}{b^{2}-a^{2}}\int _{ a}^{ b}\left (1- \frac{\sigma _{2}(r)}{\sigma _{h}}\right )^{2}r\mathrm{d}{r}. $$

This suggests that the assumed form of $\delta{\boldsymbol{\tau }}$ in equation (137) is a reasonable approximation. For a better point-wisely uniform convergence to $\sigma _{h}$, we may propose a higher order approximation to replace (137) or use either the Class 2 or Class 3 growth models shown in Sects. 10.2 and 10.3.

The corresponding temporal development of the radial residual stress $\tau _{1}$ and circumferential residual stress $\tau _{2}$ are shown in Figs. 2(b) and 2(c), respectively. It is clear that the curves of the residual stresses $\tau _{1}$ and $\tau _{2}$ demonstrate and verify the assumption of (137) that (1) the value of $\tau _{1}$ is negative in $\bar{a}<\bar{r}<\bar{b}$ while satisfying the traction-free boundary conditions at $\bar{r}=\bar{a}, \bar{b}$, (2) $\tau _{2}$ has the role of creating a uniform distribution of $\sigma _{2}$ at the end of growth by counterbalancing the initial distribution of $\sigma _{2}$ at the beginning of growth.

However, it is observed from the numerical study that although the development of residual stress by the assumption in (137) can create a uniform distribution of $\sigma _{2}$ (which is not necessarily equal to $\sigma _{h}$), it is the additional growth law for $\bar{L}_{22}\vert _{R=A}$ (or equivalently $\dot {\bar{a}}$) in equation (182) playing the role of controlling $\sigma _{2}$ to approach to the specified $\sigma _{h}$.

The $\bar{J}$ curves in Fig. 2(d) show the temporal-spatial development of the volumetric growth of the shell. It is observed that the spatial distribution of volumetric growth monotonically decreases through the shell thickness from $\bar{a}$ to $\bar{b}$. This is consistent with the distribution of the residual stress $\tau _{2}$ considered to be created by the non-uniform volumetric growth.

The temporal curves of ${\bar{a}}/A$ and ${\bar{b}}/B$ in Fig. 2(e) show the development of the inner and outer radii of the shell. It is observed that the ${\bar{a}}/A$ (or ${\bar{b}}/B$) versus $t$ curve is an indication of convergence as $\dot{\bar{a}}/A$ (or $\dot{\bar{b}}/B$) approaches to zero when the stress is achieving the homeostatic state.

Also, the dissipation increment at $R=A$, $\mathcal{D}_{A}$, may indicate the convergence. This can be observed in Fig. 7 where $\mathcal{D}_{A}$ approaches zero with respect to time $t$ showing the convergence.

### Class 2 Growth Law

The values of the model parameters in equations (143) and (183) are set as $\bar{\eta}=5.0\times 10^{-5}$ Ms^−1^ and $c_{r}=4.5\times 10^{-5}\text{ mm}$, respectively.

**Fig. 3:**
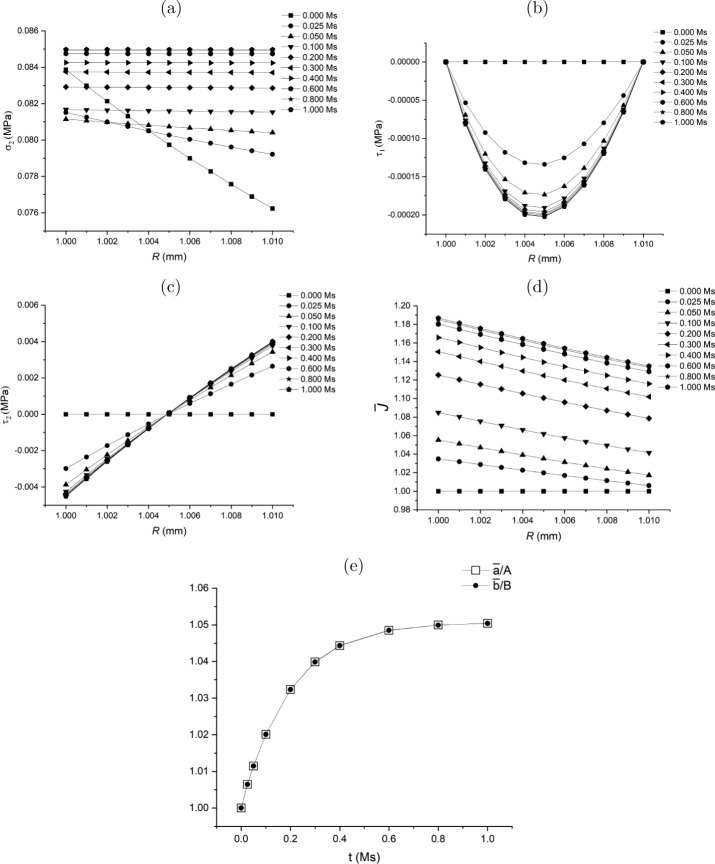
*Class 2*: The temporal development ($t\in [0,1.0]$ Ms) of (a) the circumferential stress $\sigma _{2}$, (b) the radial residual stress $\tau _{1}$, (c) the circumferential residual stress $\tau _{2}$, (d) the volumetric growth $\bar{J}$, (e) the inner and outer radii of the unloaded configuration, $\bar{a}$ and $\bar{b}$, normalised by their initial values, $A$ and $B$, respectively

By comparison to those in the Class 1 model, the results shown for the Class 2 model show a better convergence. This can be observed from Fig. 3(a) that the stress $\sigma _{2}$ shows point-wisely uniform convergence to the specified $\sigma _{h}$. The convergence is also verified by the $\bar{a}/A$ (or $\bar{b}/B$) versus $t$ curve in Fig. 3(e) and the $\mathcal{D}_{A}$ versus $t$ curve in Fig. 7.

Due to better precision and convergence, the results for the Class 2 model clearly confirm the observation mentioned for the Class 1 model that the growth law of residual stress only leads to a uniform distribution of $\sigma _{2}$ while the additional growth law of $\bar{L}_{22}\vert _{R=A}$ (or equivalently $\dot {\bar {a}}$) in equation (182) plays the role of controlling $\sigma _{2}$ to approach to the specified $\sigma _{h}$. We can observe that the growth process may be divided into two stages. Stage 1 covers a duration from $t=0$ to $t=0.1$ Ms in which $\sigma _{2}$ achieves a uniform distribution $\sigma _{2}=0.0818\text{ MPa}$ $\neq \sigma _{h}$. In stage 2 ($t\in [0.1,1.0]]$ Ms), the uniformly-distributed $\sigma _{2}$ gradually approaches and converges to the specified $\sigma _{h}$. The two stages can also be observed in the temporal development of the volumetric growth of the shell as shown in $\bar{J}$ versus $R$ curves in Fig. 3(d). Stage 1 shows a continuous increase of the absolute value of the slope of the $\bar{J}$ versus $R$ curves. By contrast, the slopes of the $\bar{J}$ versus $R$ curves in stage 2 are nearly a constant. Note that the separation of two stages may also be observed in corresponding results for the Class 1 model.

The temporal development of the radial residual stress $\tau _{1}$ and circumferential residual stress $\tau _{2}$ are shown in Figs. 3(b) and 3c, respectively. The consistency of the results in Figs. 3(b) and 3(c) with the results shown in the Figs. 2(b) and 2(c) further verifies the assumption of (137) of the Class 1 model.

It may be concluded that the Class 1 and Class 2 models show consistent behaviours. The Class 1 model provides a reasonable closed form solution while Class 2 model provides better precision and convergence.

### Class 3 Growth Law

The values of the model parameters in equations (153) and (183) are set as $\tilde{\eta}=8.8\times 10^{-6}$ Ms^−1^ and $c_{r}=4.0\times 10^{-6}\text{ mm}$, respectively.

**Fig. 4:**
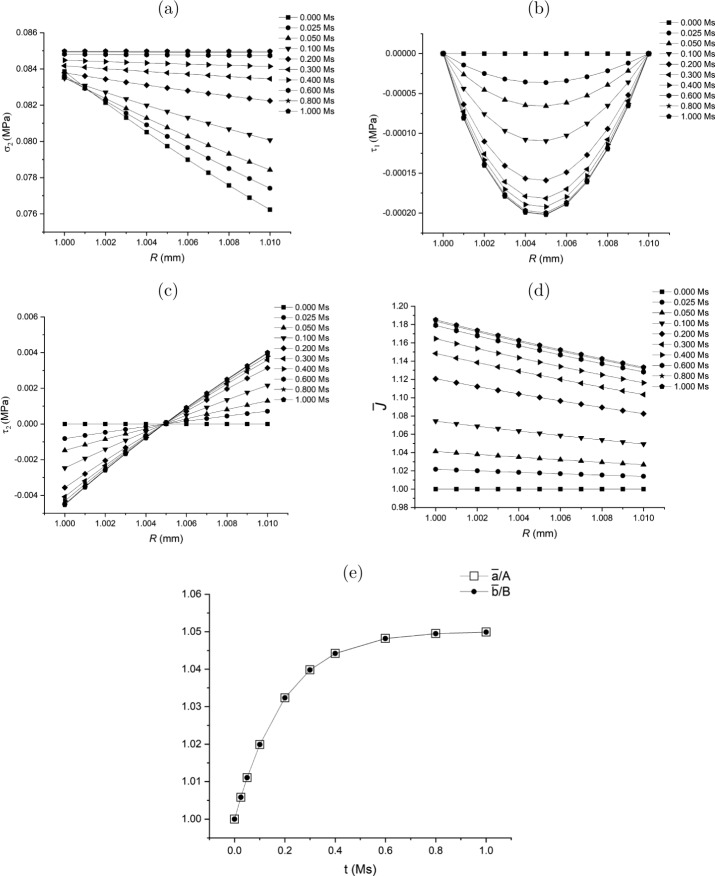
*Class 3*: The temporal development ($t\in [0,1.0]$ Ms) of (a) the circumferential stress $\sigma _{2}$, (b) the radial residual stress $\tau _{1}$, (c) the circumferential residual stress $\tau _{2}$, (d) the volumetric growth $\bar{J}$, (e) the inner and outer radii of the unloaded configuration, $\bar{a}$ and $\bar{b}$, normalised by their initial values, $A$ and $B$, respectively

Similar to the result in Class 2 model, the stress $\sigma _{2}$ in Fig. 4(a) shows point-wisely uniform convergence to the specified $\sigma _{h}$. The convergence is also verified by the $\bar{a}/A$ (or $\bar{b}/B$) versus $t$ curve in Fig. 4(e) and the $\mathcal{D}_{A}$ versus $t$ curve in Fig. 7, which is similar to the observation in the Class 2 model.

The temporal development of the radial residual stress $\tau _{1}$ and circumferential residual stress $\tau _{2}$ are shown in Figs. 4(b) and 4(c), respectively. The assumption of (137) in the Class 1 model is further verified by the consistency between the results of the Class 1 and Class 3 models.

It is interesting to observe that the results of the Class 3 model do not show the obvious separation of two stages of growth as observed in Class 1 and Class 2. The temporal development of $\sigma _{2}$ in Fig. 4(a) shows that $\sigma _{2}$ becomes point-wise uniform when $\sigma _{2}$ is very close to the specified $\sigma _{h}$ ($t=0.6$ Ms). This is also confirmed by the $\bar{J}$ versus $R$ curves of the volumetric growth shown in Figs. 4(d). Besides the specification of the model parameters, the explicit coupling between $\delta \tau _{1}$ and $\bar{L}_{22}$ as shown in the differential equations (191) may contribute to this observation of no obvious separation of the two stages.

Therefore, the numerical results indicates that Class 2 and Class 3 may be applied to different growth processes where the homeostatic states are established in different ways with respect to time.

### Class 4 Growth Law

The values of the model parameters in equations (165) and (166) are set as $n=2$ and $\eta _{c1}=-5.0\times 10^{-4}\text{ MPa}^{-1}\,\text{mm}^{-2}\,\text{Ms}^{-1}$, $\eta _{c2}=-5.0\times 10^{-4}\text{ MPa}^{-1}\,\text{mm}^{-2}$, respectively.

**Fig. 5:**
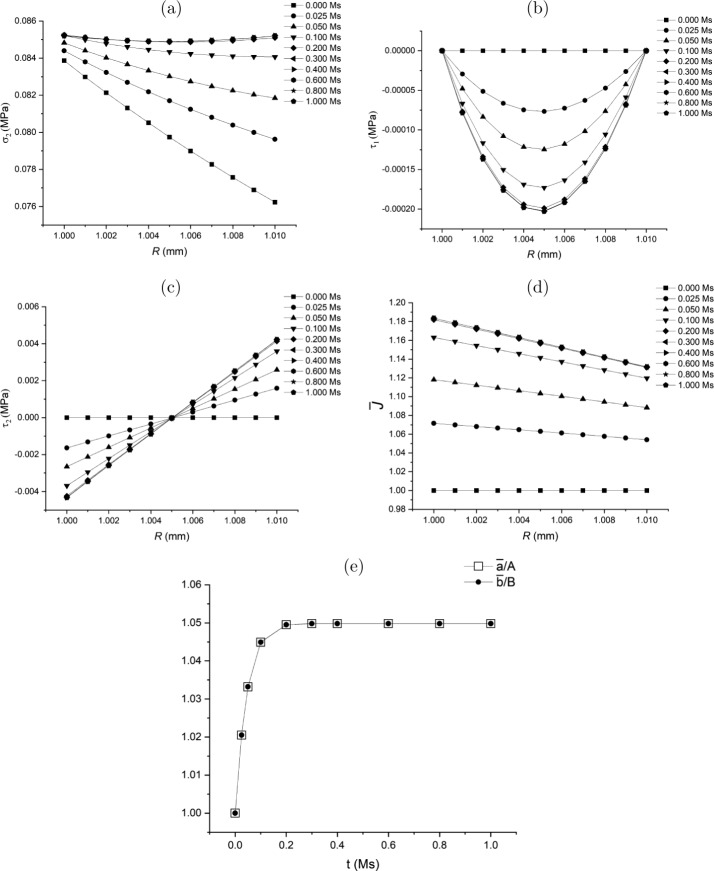
*Class 4*: The temporal development ($t\in [0,1.0]$ Ms) of (a) the circumferential stress $\sigma _{2}$, (b) the radial residual stress $\tau _{1}$, (c) the circumferential residual stress $\tau _{2}$, (d) the volumetric growth $\bar{J}$, (e) the inner and outer radii of the unloaded configuration, $\bar{a}$ and $\bar{b}$, normalised by their initial values, $A$ and $B$, respectively

Similar to the result of Class 1 model, the circumferential stress $\sigma _{2}$ approaches to the specified homeostatic stress $\sigma _{h}$ if measured by the 2-norm, $\vert \vert \sigma _{2}\vert \vert _{2}$, defined in (214) (see Fig. 5(a)). But $\sigma _{2}$ does not show a point-wise uniform distribution due to the limitation of the *assumed* analytical form of $\dot{\bar{r}}$ in equation (167).

The convergence is also verified by the $\bar{J}$ versus $R$ curves in Fig. 5(d), the $\bar{a}/A$ (or $\bar{b}/B$) ver5sus $t$ curve in Fig. 5(e), and the $\mathcal{D}_{A}$ versus $t$ curve in Fig. 7.

We observe the consistency between the results of Class 1 model and Class 4 model in the temporal development of $\tau _{1}$ and $\tau _{2}$ by the comparison between Figs. 2(b) and 2(c) and Figs. 5(b) and 5(c). This consistency between the Class 1 and Class 4 models also verifies the analytical assumption of the residual stress in equation (137) of the Class 1 model and that of $\dot{\bar{r}}$ in the equation (167) of the Class 4 model.

Note that the results for the Class 4 model do not show an obvious separation of the two stages of growth: the first stage to approach a uniform distribution of $\sigma _{2}$ and then the second stage to converge to the specified homeostatic stress $\sigma _{h}$ as shown in the results of the Class 1 model.

### Class 5 Growth Law

The value of the model parameter in equation (171) is set as ${\bar{\eta}}^{\star}=1.5\times 10^{-6}\text{ MPa}^{-1}\,\text{mm}^{-2}$.

**Fig. 6:**
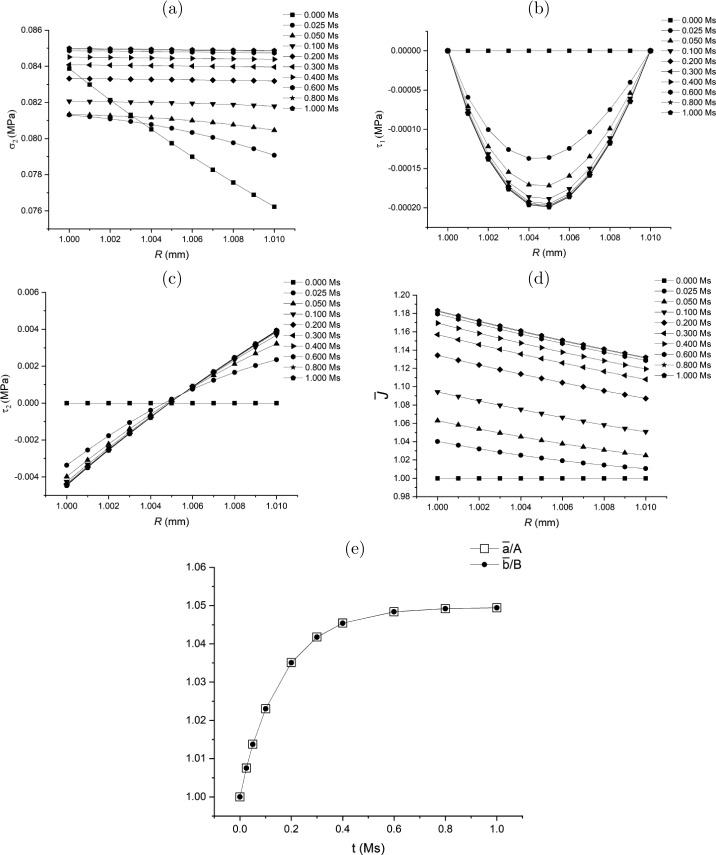
*Class 5*: The temporal development ($t\in [0,1.0]$ Ms) of (a) the circumferential stress $\sigma _{2}$, (b) the radial residual stress $\tau _{1}$, (c) the circumferential residual stress $\tau _{2}$, (d) the volumetric growth $\bar{J}$, (e) the inner and outer radii of the unloaded configuration, $\bar{a}$ and $\bar{b}$, normalised by their initial values, $A$ and $B$, respectively

It is immediately observed from the comparison of Fig. 6 and Fig. 3 that the overall behaviour of the Class 5 model is similar to that of Class 2. Both show the point-wise convergence of $\sigma _{2}$ towards the specified homeostatic state $\sigma _{2}=\sigma _{h}$, and both show the separation of the two stages of growth.

**Fig. 7:**
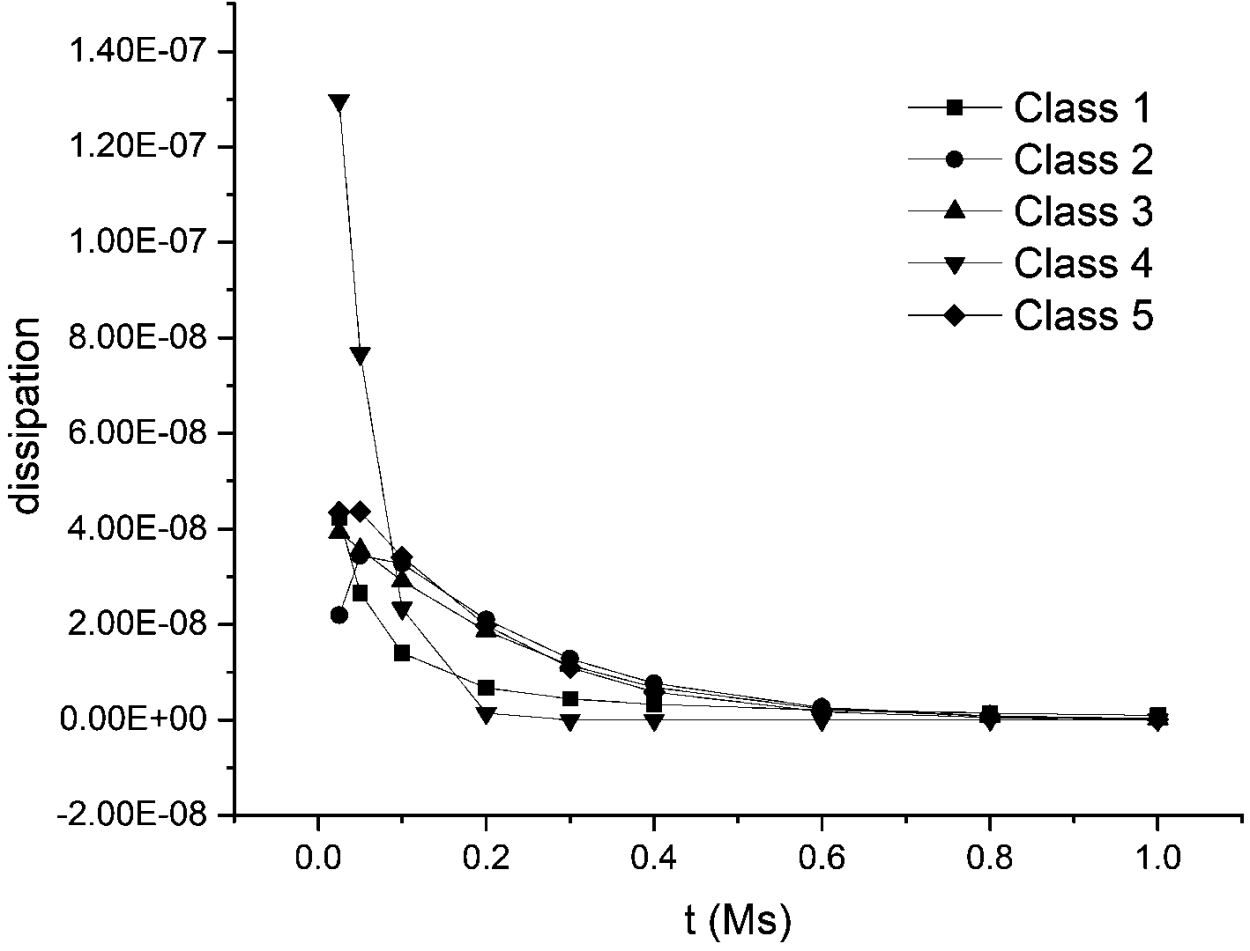
The temporal development ($t\in [0,1.0]$ Ms) of the dissipation $\mathcal{D}_{A}$ measured at the material point $A$ of the inner surface of the shell

**Fig. 8:**
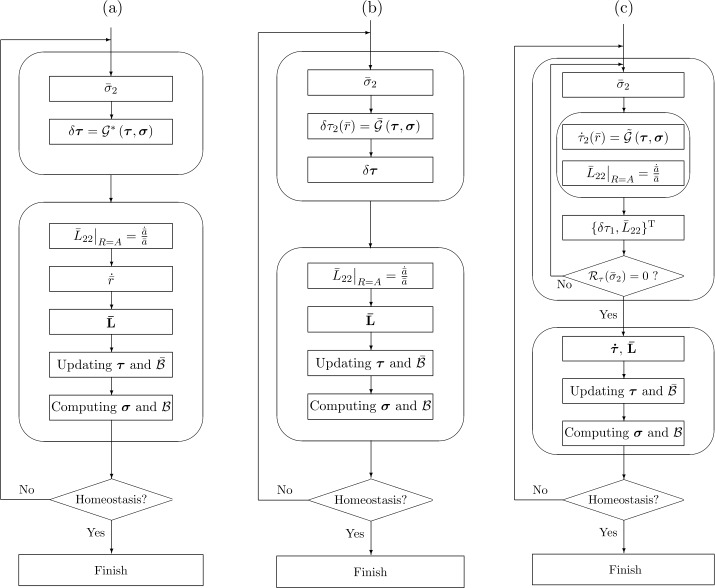
Solution flow charts for Classes 1, 2, 3 of the growth law: (a), (b), (c), respectively

**Fig. 9:**
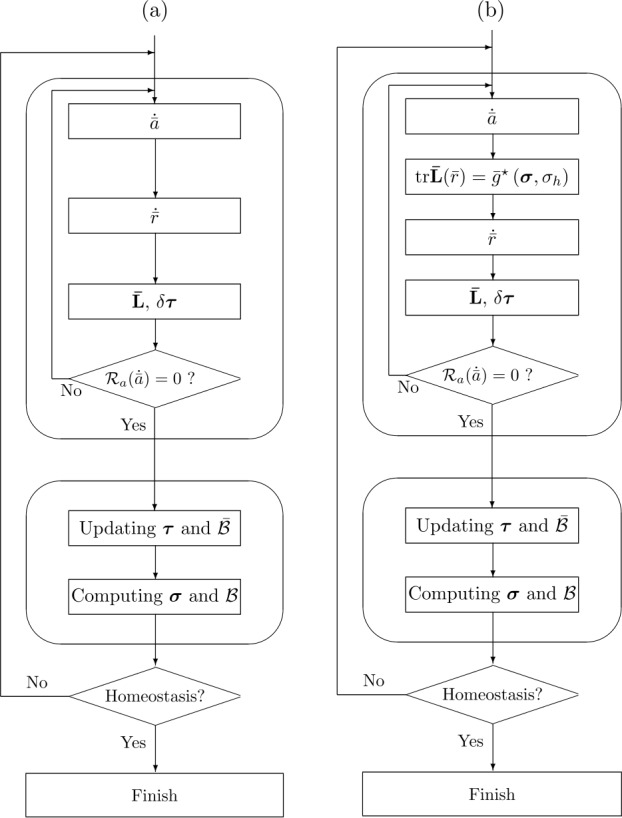
Solution flow charts for Classes 4, 5 of the growth law: (a), (b), respectively

The noticeable differences between the results of the Class 5 and Class 2 models are: the $\sigma _{2}$ versus $R$ curves in Fig. 6(a) and the $\tau _{2}$ versus $R$ curves in Fig. 6(c) of Class 5 show the stronger non-linearity in the very early stage of growth with respect to $R$ by comparison to Class 2. This difference also reflects in Fig. 7 where the $\mathcal{D}_{A}$ versus $t$ curves of the Class 5 and Class 2 models deviate from each other in the very early stage of growth but become very close to each other after the early stage, which is consistent with the overall similarity between the results of Class 5 and Class 2 models.

Moreover, the comparison between the results of the Class 5 and Class 4 models indicates some differences. The latter does not exhibit two obvious stages of growth.

### Discussion of the Numerical Results

Five classes of growth modelling are presented to fully demonstrate and validate the proposed growth modelling formulated on the unloaded configuration $\bar{\mathcal{B}}$.

The Class 1 and Class 4 models use *independently-assumed* global analytical fields of the increments of residual stress and displacement on $\bar{\mathcal{B}}$, respectively. The numerical results show that both assumed fields can converge to the specified homeostatic state. The consistency between their results as shown in Fig. 2 and Fig. 5 is a reasonable validation of both assumed analytical fields.

On the other hand, the Class 2, Class 3 and Class 5 models are formulated using local growth laws. The three classes show point-wise uniform convergence to the specified homeostatic stress, which is more precise by comparison to the global 2-norm-convergences shown in Class 1 and Class 4. This observation indicates that more sophisticated assumed fields may improve the precision of the Class 1 and Class 4 models.

Moreover, we may compare Classes 1, 2, 3 with Classes 4, 5. The former are each a kind of model containing the growth law of the increments of residual stresses for the whole domain $\bar{r}\in [\bar{a}, \bar{b}]$ and the additional growth law for $\dot{\bar{r}}$ at the location $\bar{r}=\bar{a}$. The latter are each another type of model that contain either $\dot{\bar{r}}$ or the volume increment for the whole domain $\bar{r}\in [\bar{a}, \bar{b}]$. It is observed that the results of the two kinds of models are consistent. Especially, the results of the Class 2 model are very similar to their counterparts in the Class 5 model. This consistency/similarity further validates the proposed theory and numerical methods.

We also note that the results of the five classes of models may show some different features (e.g., with or without two stages of growth). This indicates that the proposed methods can be adapted to various experimental observations.

In summary, the convergence, consistency and variety of the results shown in the case studies provide a thorough demonstration and validation of the proposed theory.

## Concluding Remarks

In the foregoing sections we have developed a theoretical framework for the analysis of residually-stressed based volumetric growth in a nonlinearly elastic solid using an incremental theory to account for the evolving growth and the accompanying residual stress. The theory has been applied to the problem of a growing spherical shell under internal pressure and illustrated for specific forms of constitutive law. Illustrations of the theory are developed by presenting the corresponding numerical methods and case studies. Detailed numerical results are provided to confirm the efficacy of the approach.

Different from the typical kinematical growth model where a growth law may be needed to assign the ‘velocity’ gradient on the *fictitious* stress-free intermediate configuration, the numerical case study of spherical shell growth demonstrates five classes of growth models formulated on the residually-stressed unloaded configuration.

What needs to be assigned for the growth laws may include: (1) increments of residual stresses, (2) increments of $\bar{r}$, and (3) the volume increment on the unloaded configuration. In principle, all of this information can be measured experimentally. This provides a direct connection between the models and experimental data/observations.

The formulation on the unloaded configuration provides a new perspective to the application of the thermodynamic constraints. It has been noticed that the typical kinematic growth models based on the intermediate configuration do not have the counterpart of the flow rules in plasticity theory that can satisfy the second law of thermodynamics *a priori*. For an interesting approach to growth using plasticity theory, we refer to [38]. In the present study, we demonstrated that it is feasible to strictly satisfy the thermodynamics constraint in the Class 1–3 growth laws *a priori*.

Moreover, the present study is able to further clarify the relation between the residual stress and the homeostatic state, bearing in mind that it is the tissue itself that drives progress towards the homeostatic state under loading. The numerical study indicates that assignment of the change of the residual stresses alone cannot achieve a specified homeostatic state. An additional control condition for $\dot{\bar{r}}$ at the inner (or outer) surface of the shell is required to allow convergence to the specified homeostatic state. This observation may shed new light into the experimental study of the growth of shell structures.

In summary, the proposed growth model provides a method to directly connect to the measurable experimental data and to integrate the key considerations of deformation, residual stresses, homeostatic state into a mathematical framework feasible to satisfy the thermodynamics constraint *a priori*.

## Data Availability

No datasets were generated or analysed during the current study.
